# Ligand and structure-based approaches for the exploration of structure–activity relationships of fusidic acid derivatives as antibacterial agents

**DOI:** 10.3389/fchem.2022.1094841

**Published:** 2023-01-06

**Authors:** Wende Zheng, Borong Tu, Zhen Zhang, Jinxuan Li, Zhenping Yan, Kaize Su, Duanyu Deng, Ying Sun, Xu Wang, Bingjie Zhang, Kun Zhang, Wing-Leung Wong, Panpan Wu, Weiqian David Hong, Song Ang

**Affiliations:** ^1^ School of Biotechnology and Health Sciences, Wuyi University, Jiangmen, China; ^2^ International Healthcare Innovation Institute, Jiangmen, China; ^3^ School of Biomedicine and Pharmaceutical Sciences, Guangdong University of Technology, Guangzhou, China; ^4^ The State Key Laboratory of Chemical Biology and Drug Discovery, Department of Applied Biology and Chemical Technology, The Hong Kong Polytechnic University, Hung Hom, Kowloon, Hong Kong SAR, China

**Keywords:** fusidic acid, derivatives, pharmacophore model, antibacterial, structure–activity relationships

## Abstract

**Introduction:** Fusidic acid (**FA**) has been widely applied in the clinical prevention and treatment of bacterial infections. Nonetheless, its clinical application has been limited due to its narrow antimicrobial spectrum and some side effects.

**Purpose:** Therefore, it is necessary to explore the structure–activity relationships of **FA** derivatives as antibacterial agents to develop novel ones possessing a broad antimicrobial spectrum.

**Methods and result:** First, a pharmacophore model was established on the nineteen **FA** derivatives with remarkable antibacterial activities reported in previous studies. The common structural characteristics of the pharmacophore emerging from the **FA** derivatives were determined as those of six hydrophobic centers, two atom centers of the hydrogen bond acceptor, and a negative electron center around the C-21 field. Then, seven **FA** derivatives have been designed according to the reported structure–activity relationships and the pharmacophore characteristics. The designed **FA** derivatives were mapped on the pharmacophore model, and the Qfit values of all **FA** derivatives were over 50 and **FA-8** possessed the highest value of 82.66. The molecular docking studies of the partial target compounds were conducted with the elongation factor G (EF-G) of *S. aureus*. Furthermore, the designed **FA** derivatives have been prepared and their antibacterial activities were evaluated by the inhibition zone test and the minimum inhibitory concentration (MIC) test. The derivative **FA-7** with a chlorine group as the substituent group at C-25 of **FA** displayed the best antibacterial property with an MIC of 3.125 µM. Subsequently, 3D-QSAR was carried on all the derivatives by using the CoMSIA mode of SYBYL-X 2.0.

**Conclusion:** Hence, a computer-aided drug design model was developed for **FA**, which can be further used to optimize **FA** derivatives as highly potent antibacterial agents.

## 1 Introduction

Fusidic acid (FA), a typical antibiotic with excellent bioactivity against *Staphylococcus aureus* including the strain that produced cross resistance with other antibiotics, has been applied in clinical therapy since the 1960s (Collignon and Turnidge, 1999; Turnidge, 1999). The study on the antibacterial mechanism of **FA** showed that the elongation factor G (EF-G) of the bacteria was interfered and the production of bacterial proteins was inhibited (Tanaka et al., 1968; Bodley et al., 1969). Accordingly, the relevant protein of the EF-G has always been implemented as a target acceptor in the development of **FA**-type antibiotics (Borg et al., 2015; Belardinelli and Rodnina, 2017; Lu et al., 2019). However, the narrow antibacterial spectrum of **FA**, which merely possessed the activity against Staphylococci, limited its practical application in extensive medical treatment (Petrosillo et al., 2018). Therefore, it became increasingly important to design and synthesize new **FA** derivatives to explore a broad range of relationships between structures and antibacterial activity. According to the literature, the structure–activity relationships (SARs) between **FA** derivatives and antibacterial activity have been studied (Godtfredsen et al., 1965; Von Daehne et al., 1979; Duvold et al., 2001). The reported SAR demonstrated that the hydroxyl group at C-3 played a crucial role in drug activity. As a recent study showed blocking the metabolic sites (21-COOH and 3-OH) of **FA** and its derivatives could maintain the antibacterial activity with a prolonged half-life (Lu et al., 2019). Moreover, it has been reported that the hydroxylation at C-27 of **FA** and its derivatives could significantly cause the vanishment of the antibacterial activity (Ragab et al., 2020). Hence, the further SARs of **FA** should be obtained through more designed derivatives and their bioassay tests.

Nowadays, computer-aided drug design (CADD) has become an integral component involving drug discovery and development since it has enormous leverage as an auxiliary tool to raise economic efficiency and reduce time costs (Cerqueira et al., 2015). Advanced rational design techniques combined with computational methodologies have been utilized to create more effective and creative medications (Fjell et al., 2012; Cardoso et al., 2019). The rational design of innovative pharmaceuticals, with the aim of creating pharmaceutical products with more specificity by calculated simulation, has emerged as a crucial aspect of medicinal chemistry (Mouchlis et al., 2020). Pharmacophore-based and docking-based screening are two classic CADD approaches, which were usually applied in virtual screening to select the potential bioactive derivatives (Niu et al., 2012; Sangeetha et al., 2017). Recently, the discovery of a novel drug has benefited greatly from the use of pharmacophore-based virtual screening (PBVS), especially when there is a lack of information regarding the three-dimensional structure of the desired protein target (Sharma et al., 2020; Zhu et al., 2020). In addition, the investigation of the comparison showed that the result of the pharmacophore-based method had higher accuracy than the docking-based method in the experiment (Chen et al., 2009; Talambedu et al., 2017).

In this study, a pharmacophore model has been constructed to design the **FA** derivatives and molecular docking was used to predict the interactions between **FA** derivatives and the target protein EF-G. The antibacterial activities of the **FA** derivatives were assessed by the inhibition zone test and the MIC assay. Furthermore, the quantitative structure–activity relationships (QSARs) of **FA** were investigated with a thorough inquiry according to biological test data. All in all, this study has provided a novel pharmacophore model to select antibacterial **FA** derivatives and studied the relationship between the structures and bioactivity.

## 2 Results and discussion

### 2.1 Establishment of a pharmacophore model

**FIGURE 1:**
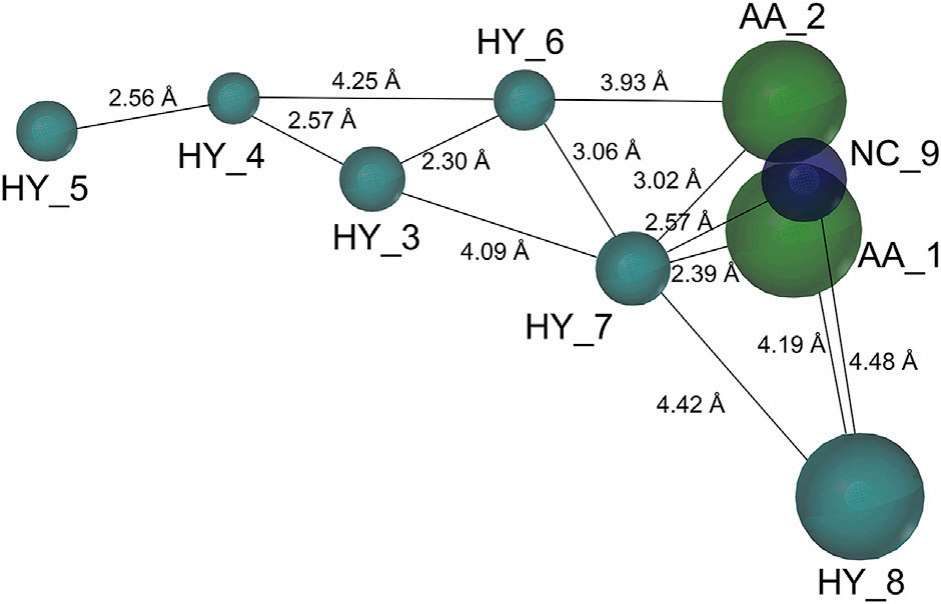
Map of a common feature pharmacophore model. AA, the atom center of the hydrogen bond acceptor; HY, hydrophobic group; and NC, negative center.

Based on a set of **FA** derivatives with remarkable antibacterial activity reported in previous studies (Godtfredsen et al., 1966; Riber et al., 2006; Lv et al., 2017; Kong et al., 2018; Salimova et al., 2018; Singh et al., 2020), a pharmacophore model was established to gain an insight into the necessary features for designing antibacterial agents. A total of 19 **FA** derivatives selected from the literature reports were aligned by using the GALAHAD module of SYBYL-X 2.0. The assessment parameters generated by two similar models are shown in Table 1, including data of specificity, N-hits, feats, energy, sterics, H-bond, and Mo-Qry. The specificity data of the model, which is a logarithmic indicator of the expected discrimination of each query, are determined by the number of features they contain and the extent of dissociation. Identical specificity values of 5.70 indicated that the two models could come to an anticipant result. Moreover, the model had nine pharmacophore features, i.e., six hydrophobic centers (HYs), two H-bond acceptors, and a negative center (NC) (Figure 1). The hydrophobic centers were distributed in the indole ring and the FA skeleton frame aromatic ring, two H-bond acceptors were found in the carbonyl group and the ester group, and the negative center was distributed in the carboxyl group of the FA derivatives at C-21, which sketched the common structural characteristics of pharmacophore emerging from the FA derivatives with antibacterial activity.

**TABLE 1:** Assessment parameters of the pharmacophore theory produced by the GALAHAD module.

Model	Specificity	N-hits	Feats	Energy	Sterics	H-bond	Mo-Qry
1	5.70	19	9	21.28	24,609.90	741.20	169.84
2	5.70	19	9	21.28	24,609.90	741.20	169.84

N-hits, actual number hit; feats, total number of features in the model query; energy: the total energy of the model; sterics, steric overlap for the model; H-bond, pharmacophoric concordance; Mo-Qry, the agreement between the query tuplet and the pharmacophoric tuplet for the ligands as a group.

### 2.2 Design and validation of the derivatives

Seven **FA** derivatives were conceived according to the reported structure–activity relationships and the characteristics of the pharmacophore model by modifying the C-3, C-21, and C-25 positions of **FA**. The designed **FA** derivatives were validated by analyzing the matching degree with the pharmacophore model through the Qfit value. The values of all derivatives were over 50 and **FA-8** possessed the highest Qfit value of 82.66, which indicated that the design of the derivative was reasonable and the designed **FA** derivatives possessed potential antimicrobial activity. The structures, molecular surface lipophilic potential photographs, and Qfit values of derivatives are shown in Table 2. There was still high hydrophobic potential maintained in the C-25 position when methyl was converted into an object of low-size profile, such as hydrogen, chlorine, and bromine. Additionally, strong negative electrical potential in the C-21 position field was not significantly altered by the creation of the lactonic ring. The aim of modification at C-3 was to maintain and even strengthen the lipophilic tendency within this range of the **FA** skeleton frame.

**TABLE 2:** Structure of the designed derivatives and the Qfit values.

Compound	Chemical structure	Molecular surface lipophilic potential	Qfit
**FA-6**	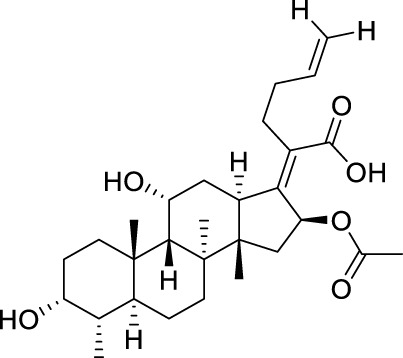	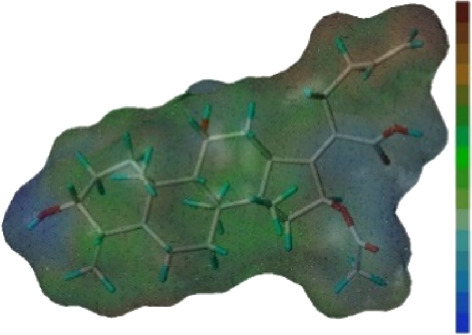	81.83
**FA-7**	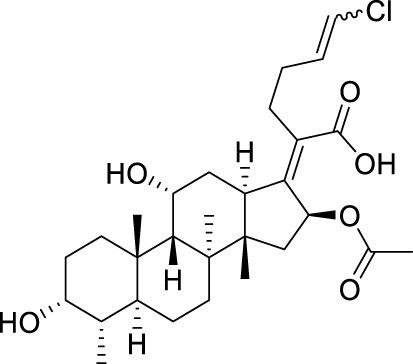	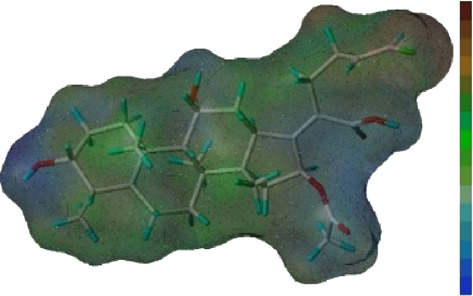	81.92
**FA-8**	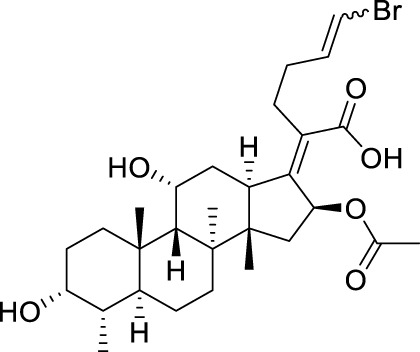	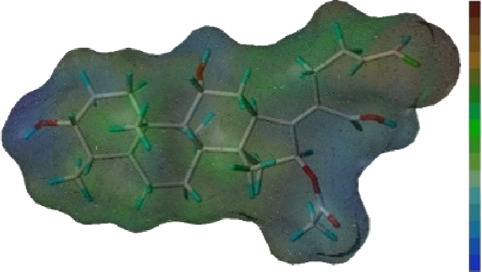	82.66
**FA-9**	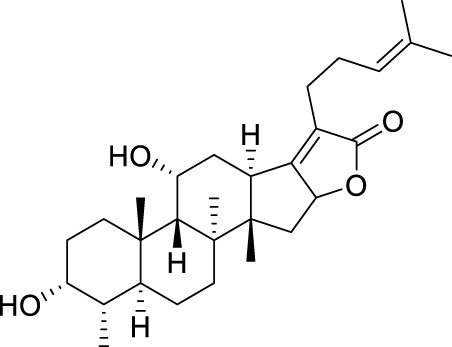	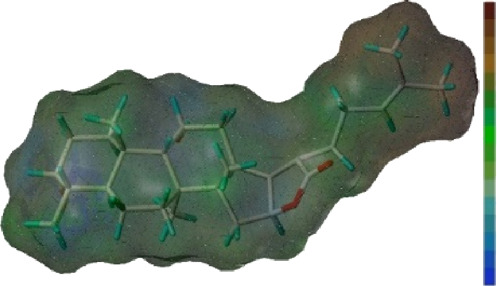	54.45
**FA-20**	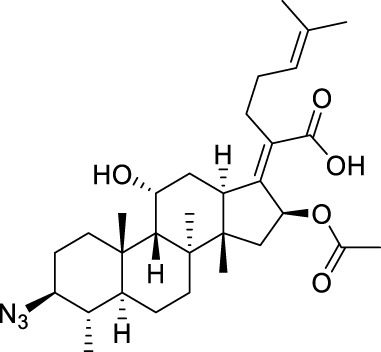	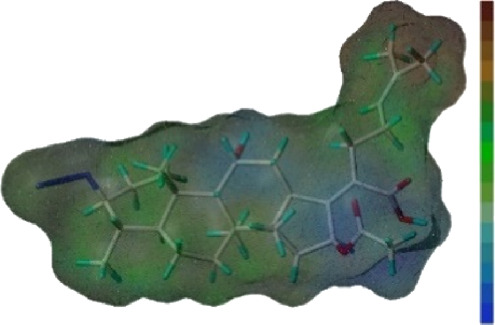	79.98
**FA-22**	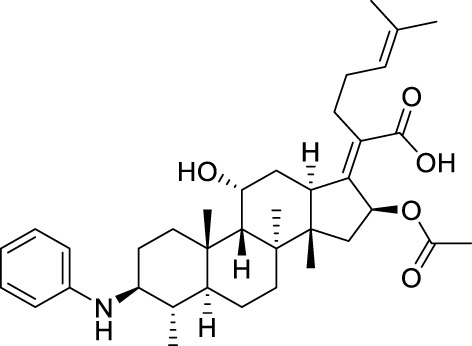	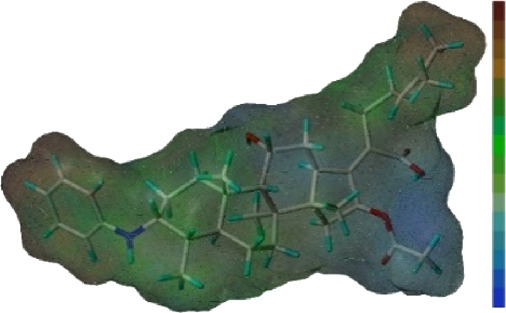	50.67
**FA-24**	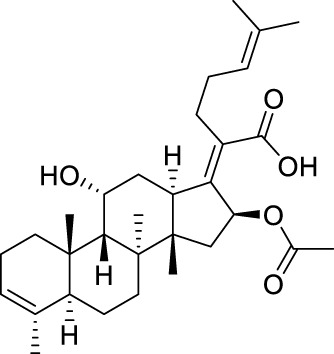	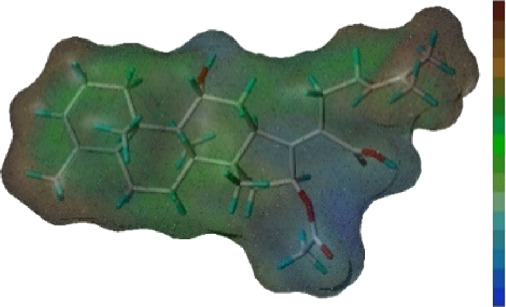	56.29

### 2.3 Molecular docking

**FIGURE 2:**
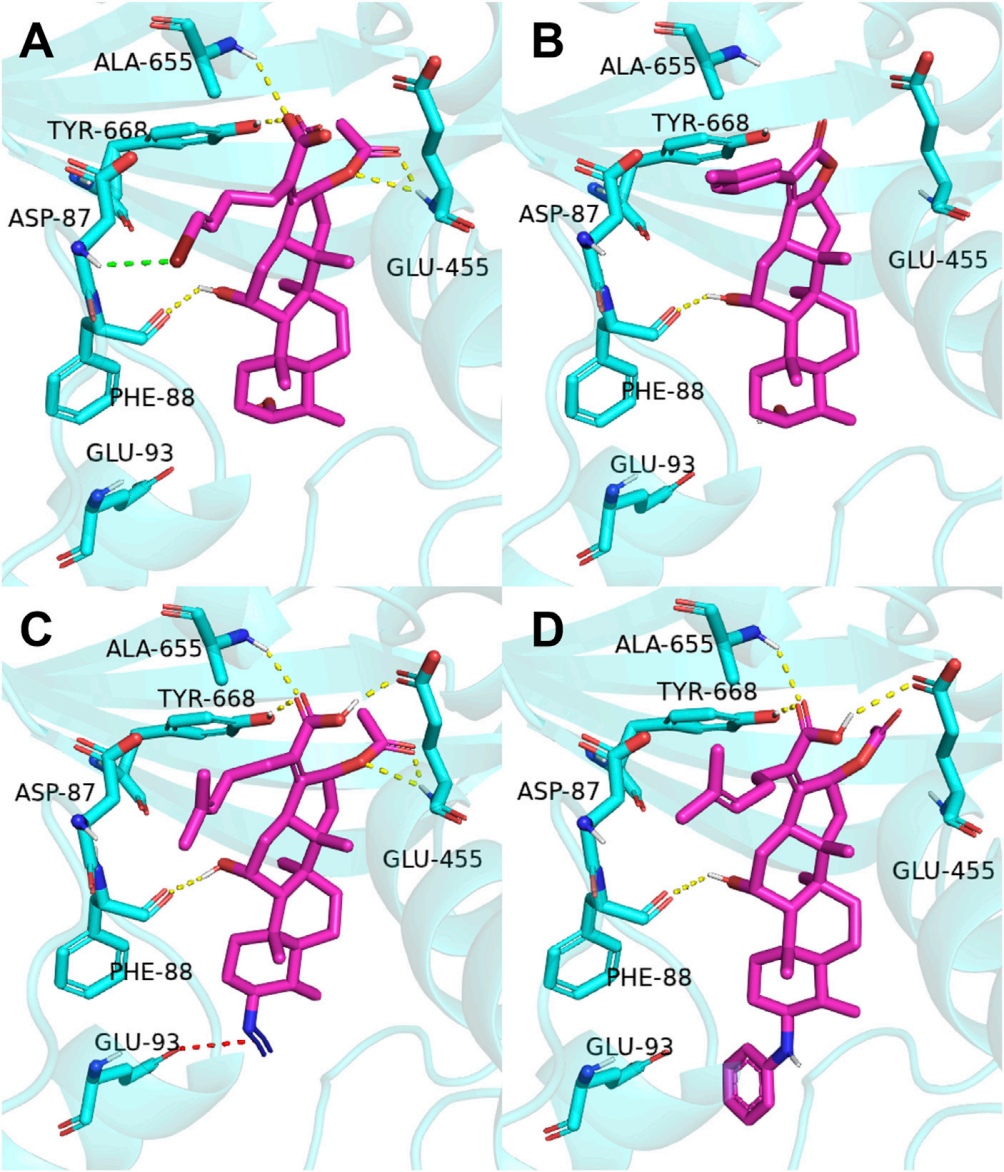
Binding mode of four derivatives in the *S. aureus* EF-G pocket: **(A) FA-8**; **(B) FA-9**; **(C) FA-20;** and **(D) FA-22**. The relevant ligand molecules were colored by magenta, and the vital amino acid was colored by cyan. The red color dash indicated the salt bridge force; the yellow color dash indicated the hydrogen bond; and the green color indicated the halogen bond interaction.

The previous study reported that **FA** was considered as an antibiotic by interfering with the EF-G of *S. aureus* (Chen et al., 2010). Therefore, it was applied therapeutically to treat Gram-positive bacterial infections, such as *S. aureus* (Lannergård et al., 2009). As shown in Figure 2, the target derivatives **FA-8**, **9**, **20**, and **22** were docked to the EF-G to investigate the action between the molecular and the receptor protein. The results showed that the interactions between the carboxyl groups of **FA** derivatives and the binding pockets existed. Compound **FA-8** could engender good affinities to Ala655, Tyr668, Glu455, and Phe88 in the active site by a hydrogen bond (Figure 2A). This kind of action of the hydrogen bond existed likewise in the mode of **FA-20** and **FA-22** fitting to the protein pocket (Figures 2C, D). However, FA-9 docking results saw a massive loss of these key interactions (Figure 2B), which corresponded to the low pharmacophore score. In addition, brominated **FA-8** had a halogen bond with Asp87, and the azide group in **FA-20** formed a salt bridge with Glu93, which may be positive features to obtain better activities. The results illustrated that the molecular docking model and the pharmacophore model could not be unanimous. It was not surprising that many epactal interactions, such as hydrogen bond and halogen bond interactions, predicted by the binding model may well compensate for some losses of key interactions.

### 2.4 Chemistry

**SCHEME 1:**
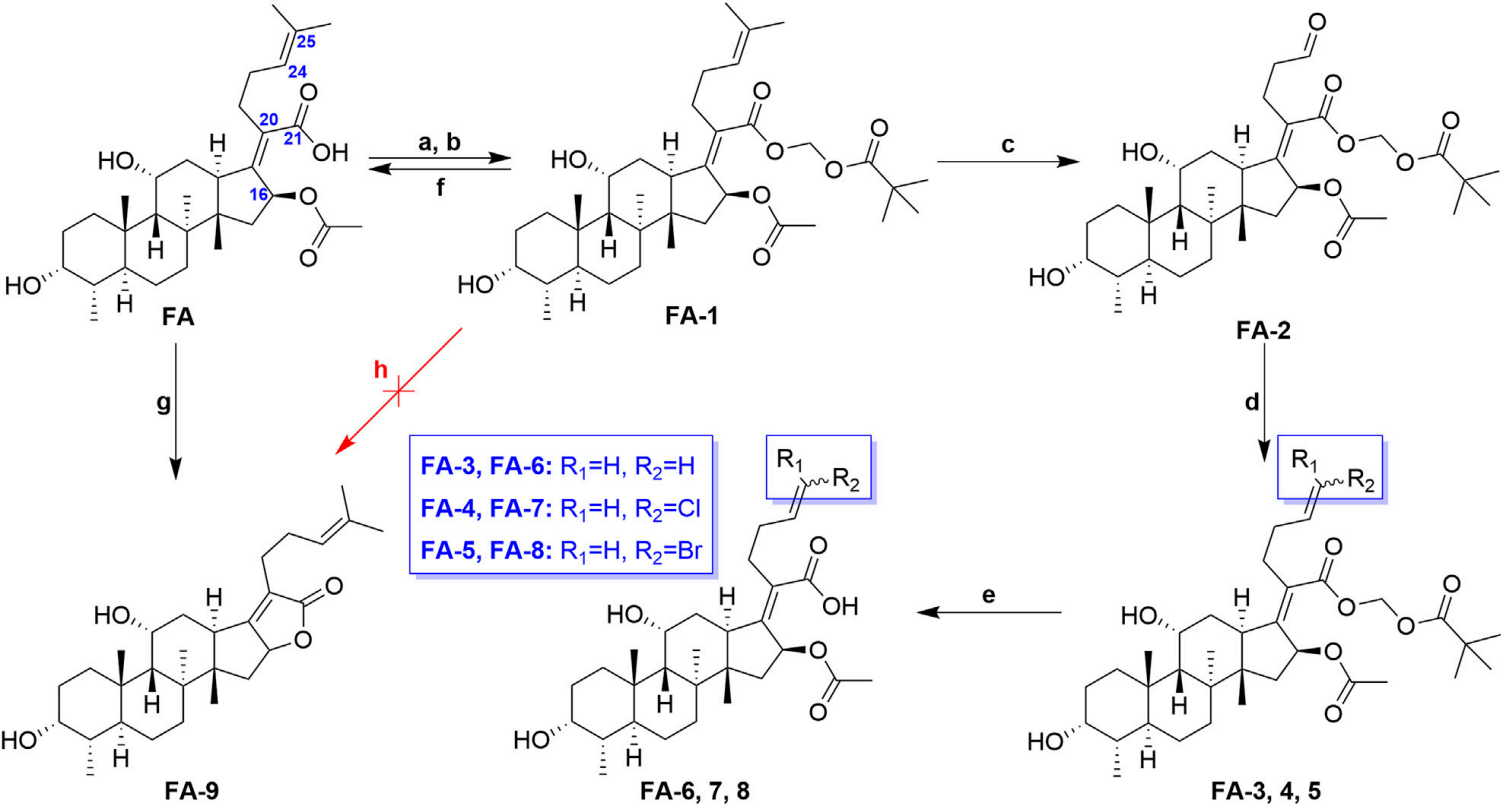
Synthetic route of **FA-6**∼**9**. Reagents and conditions: **(A)** DMF, Et_3_N (1.3 eq.), 30 min; **(B)** tBuCO_2_CH_2_Cl, overnight, 50°C; **(C)** O_3_, NMO (1 eq.), DCM, 0°C; **(D)** Wittig’s reaction; **(E)** K_2_CO_3_ (2 eq.), methanol, r. t., 1 h; **(F)** K_2_CO_3_ (2 eq.), methanol, r. t., overnight; **(G)** 2 N NaOH (5 eq.), ethanol, refluxed, overnight; and **(H)** K_2_CO_3_ (2 eq.), methanol, r. t., overnight.

The previous studies put forward some enlightenment that the modification at C-25 of **FA** could be beneficial for maintaining the antibacterial effect (Riber et al., 2006; Zhao et al., 2013). A group of target **FA** derivatives (**FA-6** to **FA-8**) was synthesized, as shown in Scheme 1. The C-25 position of **FA** had been modified successfully according to the literature reports; however, there were very few modifications with simple and small-sized atoms with functional characteristics at this position that have been carried out to estimate the antibacterial activity. We have commenced with this route by preparing several vital intermediate **FA** derivatives. The **FA** triethylamine, as an acid-binding agent, and chloromethyl pivalate were dissolved in DMF and stirred overnight at 50°C. Thus, the **FA-1** was procured with protected carboxyl groups. The **FA-2** was produced by oxidation of **FA-1** with N-methyl morpholine N-oxide (NMO) in the presence of ozone at 0°C (Schwartz et al., 2006). The derivatives **FA-6**, **FA-7**, and **FA-8** were obtained through the next simple steps such as Wittig’s reaction and de-esterification. To clarify the stability of the ester group at C-16 under different alkaline conditions, potassium carbonate and sodium hydroxide were used to promote the lactone reaction of **FA** and the intermediate **FA-1,** respectively. As a result, the lactone derivative **FA-9** was generated by the esterification of **FA** with sodium hydroxide. The syntheses of the derivatives (**FA-6∼9**) are outlined in Scheme 1. The new **FA** derivatives were determined by using NMR, HRMS, and CHNS-O elemental analyzer.

**SCHEME 2:**
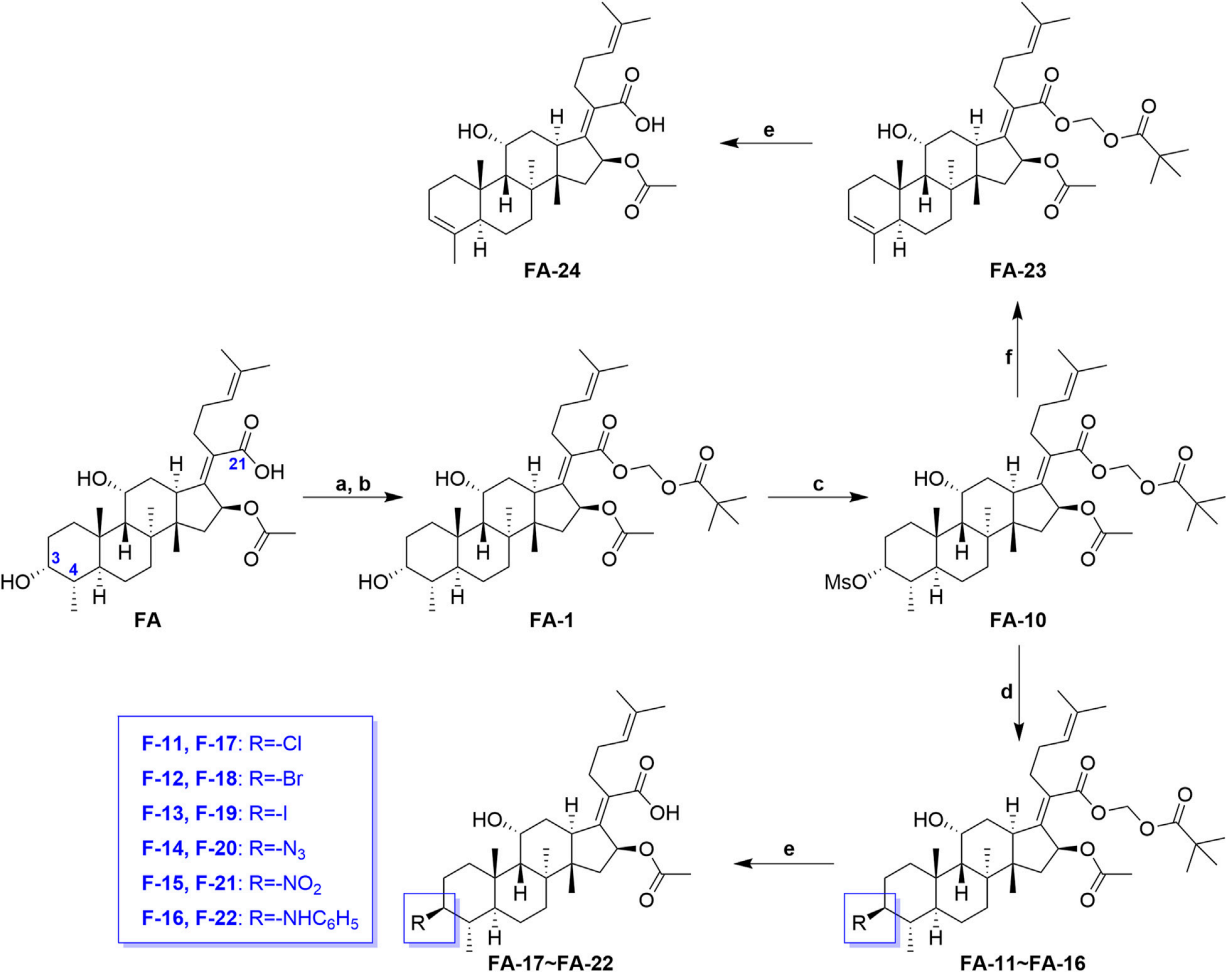
Synthetic route of **FA-17∼22** and **FA-24**. Reagents and conditions: **(A)** DMF, Et_3_N (1.3 seq.), 30 min; **(B)** tBuCO_2_CH_2_Cl, overnight, 50°C; **(C)** pyridine (2 eq.), methane sulfonyl chloride (5 eq.), DCM, overnight; **(D)** tetrabutylammonium chloride/bromide/iodide/nitrite/sodium azide (2 eq.), THF, reflux, overnight; **(E)** K_2_CO_3_ (2 eq.), methanol, r. t., 1 h; **(F)** 2,6-lutidine, 130°C, 2 h.

Scheme 2 shows the syntheses of FA-17∼22 and FA-24. Briefly, FA-1 was treated with the methane sulfonyl chloride and the pyridine in dichloromethane and afforded product FA-10. Subsequently, on one hand, the methane sulfonyloxy in FA-10 was replaced by the azide group, phenylamino group, and halogens to afford FA-11∼16, respectively. On the other hand, methane sulfonyloxy was reduced into a double bond in the positions of C-3 and C-4 to give FA-23. Finally, all the culminating products (FA-17∼22 and FA-24) were obtained by deblocking the protected ester at the C-21 position with potassium carbonate as the base reagent according to the ester stability experiment of FA derivatives. In this procedure, the related derivatives were identified mainly by HRMS.

### 2.5 Biological evaluation

#### 2.5.1 Inhibition zone test

As shown in Table 3, the antibacterial activities of the **FA** derivatives were assessed using the inhibition zone test. Compound **FA-6** possessed remarkable activity against Gram-positive germs with the corresponding inhibition zone diameters of 18.89 ± 0.03, 19.72 ± 0.12, and 14.96 ± 1.21 mm in a relatively low dosage (0.83 nmol). As the dosage increased, **FA-17**∼**22** and **FA-24** displayed obvious inhibition zones against Gram-positive bacteria. However, there was no sign for all target derivatives to inhibit Gram-negative germs in this test.

**TABLE 3:** The inhibition zone test of FA and its derivatives against bacterial strains.

Compound	Dosage (nmol)	Diameters of inhibition zones (mm)^a^				
		*Staphylococcus aureus* ATCC 6538	*Staphylococcus albus* ATCC 29213	*Staphylococcus epidermidis* ATCC 12228	*Escherichia coli* CMCC 44102	*Salmonella typhimurium* CMCC 50115
**FA**	0.12	20.31 ± 0.34	19.43 ± 0.42	19.91 ± 0.20	<6	<6
**FA-1∼2**	100	<6	<6	<6	<6	<6
**FA-3∼5**	—^b^	—	—	—	—	—
**FA-6**	0.83	18.89 ± 0.03	19.72 ± 0.12	14.96 ± 1.21	<6	<6
**FA-7**	5	26.00 ± 1.24	24.64 ± 0.56	25.89 ± 0.99	<6	<6
**FA-8**	5	25.68 ± 1.04	25.84 ± 0.88	27.27 ± 0.81	<6	<6
**FA-9∼16**	25	<6	<6	<6	<6	<6
**FA-17**	25	13.63 ± 0.10	16.91 ± 0.38	15.38 ± 0.60	<6	<6
**FA-18**	25	19.65 ± 0.05	21.69 ± 0.27	20.49 ± 0.50	<6	<6
**FA-19**	25	18.55 ± 1.86	18.79 ± 1.23	19.25 ± 0.68	<6	<6
**FA-20**	25	11.55 ± 0.02	12.88 ± 0.21	10.19 ± 0.57	<6	<6
**FA-21**	25	9.63 ± 0.45	9.20 ± 0.26	8.05 ± 0.21	<6	<6
**FA-22**	25	14.42 ± 1.32	16.80 ± 1.23	12.22 ± 0.89	<6	<6
**FA-23**	—	—	—	—	—	—
**FA-24**	25	12.64 ± 1.16	15.56 ± 0.89	16.23 ± 0.98	<6	<6
**Gatifloxacin**	1	19.12 ± 0.73	17.13 ± 0.64	18.67 ± 0.25	NT^c^	NT

^a^ No diameter of diffusion was determined.

^b^ Not detected.

^c^ Not tested. Gatifloxacin was used as a positive control.

#### 2.5.2 The minimum inhibitory concentration (MIC) test

Thenceforward, the MIC test was carried out to evaluate the antibacterial effect of the **FA** derivatives. As shown in Table 4, the C-25 positions of the **FA** derivatives were altered chemically with the halogen and hydrogen groups and the derivatives maintained the bioactivity against Gram-positive bacteria. **FA-7** with a chlorine group as the substituent group at C-25 displayed the best medicinal property with a MIC of 3.125 µM. None of the intermediates showed any antibacterial activity in this assay. Simultaneously, it was noteworthy that esterification at C-21 resulted in the complete loss of activity. Therefore, the integrity of carboxyl at C-21 was indispensable for the preservation of activity. Additionally, the results of the bioassay showed that **FA-20**, **FA-21**, and **FA-22** possessed weaker antimicrobial activity than those owned by the halogen groups. Therefore, the existence of the halogen groups was much more conducive to antibacterial activity.

**TABLE 4:** MICs of FA and its derivatives against the bacterial strains.

Compound	MICs (µM)^a^				
	*S. aureus* ATCC 6538	S. *albus* ATCC 29213	S. *epidermidis* ATCC 12228	*E. coli* CMCC 44102	*S. typhimurium* CMCC 50115
**FA**	3.125	3.125	3.125	>200	>200
**FA-1∼FA-2**	>200	>200	>200	>200	>200
**FA-3∼FA-5**	—^b^	—	—	—	—
**FA-6**	20.84	10.41	20.84	>200	>200
**FA-7**	6.25	3.125	3.125	>200	>200
**FA-8**	12.5	12.5	6.25	>200	>200
**FA-9∼16**	>200	>200	>200	>200	>200
**FA-17**	25	12.5	25	>200	>200
**FA-18**	25	6.25	6.25	>200	>200
**FA-19**	25	12.5	25	>200	>200
**FA-20**	100	100	100	>200	>200
**FA-21**	100	100	100	>200	>200
**FA-22**	100	50	50	>200	>200
**FA-23**	>200	>200	>200	>200	>200
**FA-24**	50	25	50	>200	>200
**Gatifloxacin**	0.2	0.2	0.2	NT^c^	NT

^a^ MIC values in the experiment were performed in triplicate.

^b^ Not detected.

^c^ Not tested. Gatifloxacin was used as a positive control.

### 2.6 Quantitative structure–activity relationship (QSAR)

**FIGURE 3:**
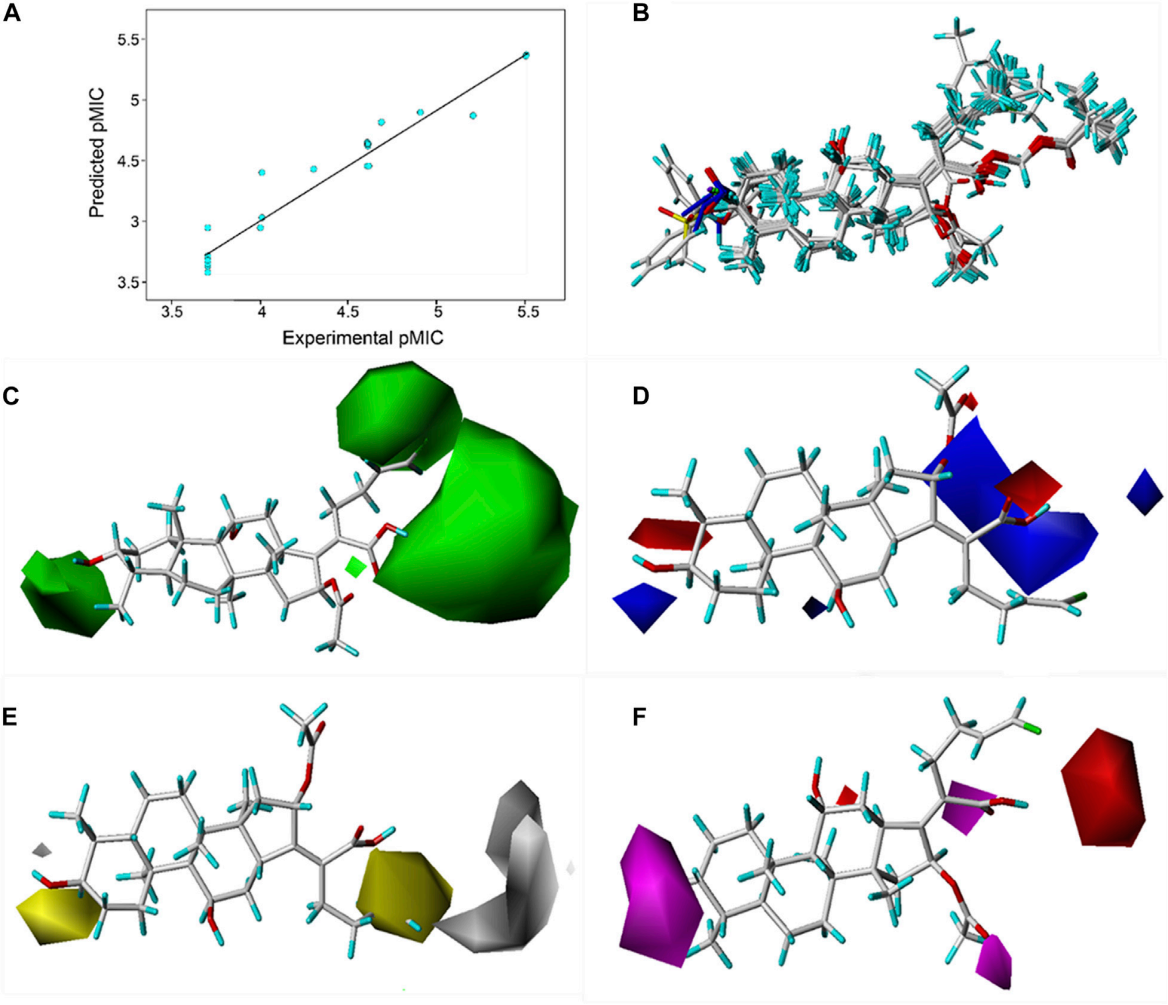
**(A)** Relationship between the experimental and predicted antibacterial activities by the QSAR model, pMIC = -lg MIC, showing *R* ^2^ = 0.921; **(B)** flexible alignment of derivatives shown in the stick model; **(C)** steric fields, green favorable and yellow disfavored; **(D)** Phase electrostatic fields, blue favorable and red disfavored; **(E)** hydrophobic fields, yellow favorable and white disfavored; and **(F)** H-bond acceptor fields, magenta favorable and red disfavored.

Based on the pMIC (negative logarithm of the MIC) values of the synthesized **FA** derivatives, a comparative molecular similarity index analysis (CoMSIA) model was constructed to explore the structure–activity relationship of the constructed **FA** derivatives against *S. aureus*. Cross-validated coefficients (q2), non-cross-validated correlation coefficients (*r* ^2^), standard error of estimates, and F-test values F) were 0.55, 0.921, 0.167, and 110.547 in the constructed CoMSIA model, respectively. The obtained q^2^ and *r* ^2^ values were in the range of the internal validations (q^2^ > 0.5 and *r* ^2^ > 0.8), which indicated that the predictive accuracy of the constructed 3D-QSAR models was credible. The results displayed a linear relationship between the experimental and predicted values as shown in the scatter plot (Figure 3A). As shown in Figure 3B, the aligned compounds were imported into Phase to make partial least-squares (PLS). The model was then used to correlate the activities of these compounds with the Phase field data calculated from their 3D structures. The steric contour map of CoMSIA is given in Figure 3C, and the result suggested that the larger the size of substituents at C-3, C-21, and C-25 positions, the stronger will be the antibacterial activity of derivatives. The electrostatic contour map in Figure 3D shows that the introduction of the atom with high electrostatic potential at C-21 and C-25 would be beneficial to improve the antibacterial activity of derivatives. In addition, the hydrophobic group and the group of hydrogen bond acceptors at C-3 and C-21 positions would contribute to enhanced antibacterial activities, as shown in Figures 3E, F. Overall, the presences of halogen groups at the positions C-3 and C-25 were more advantageous for maintaining antibacterial activity than **FA**.

## 3 Materials and methods

### 3.1 Generation of the pharmacophore model and design of derivatives

The pharmacophore model was constructed using the Genetic Algorithm with Linear Assignment of Hypermolecular Alignment of Database (GALAHAD) module of SYBYL-X 2.1 software (Tripos Inc., St. Louis, MO, United States), and two similar models with varied parameters including specificity, N-hits, feats, and energy were first generated by setting 2, 5, and 4 for the parameters of population size, maximum generation, and mols, respectively. The pharmacophore model that was suitable for screening should basically meet the following requirements: specificity >4, N-hits (the number of compounds used for the construction), and relatively low energy that indicated stability. A decoy set method was then applied to evaluate the quality of the model. The decoy set in this study was composed of 19 **FA** derivatives with notable antibacterial activities taken from the published literature reports, as shown in Table 5. Following the creation of the pharmacophore models, the most effective model was carried out and a 3D search query was applied for the designed derivatives. Then, a column of Qfit parameters was loaded with the **FA** derivatives. Qfit is a value between 0 and 100, where 100 is the best. It represents how close the ligand atoms of the compounds match the query target coordinates. Meanwhile, the Qfit values for derivatives were shown to assess the degree of correlation with antibacterial activity. In this study, the minimum standard value of Qfit was first set to 50, and seven compounds with Qfit values of more than 50 were obtained.

**TABLE 5:** Known FA derivatives with notable antibacterial activities used to generate a pharmacophore model.

Chemical structure	MIC (reference)	Chemical structure	MIC (reference)
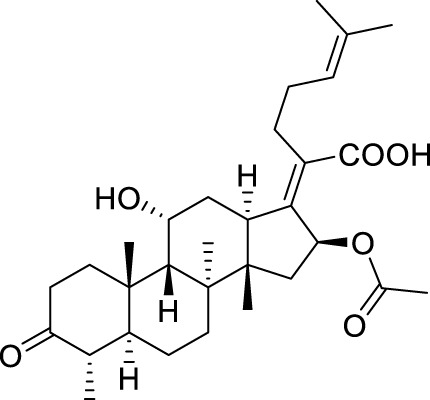	1 μg/mL (Garcia Chavez et al., 2021)	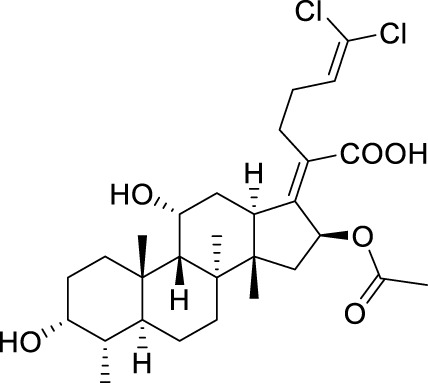	0.25 μg/mL (Garcia Chavez et al., 2021)
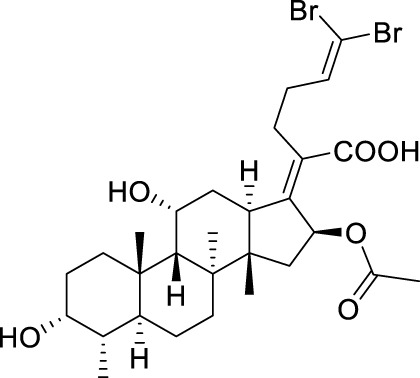	0.125 μg/mL (Garcia Chavez et al., 2021)	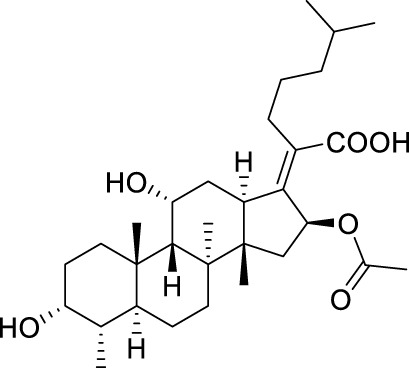	0.25 μg/mL (Garcia Chavez et al., 2021)
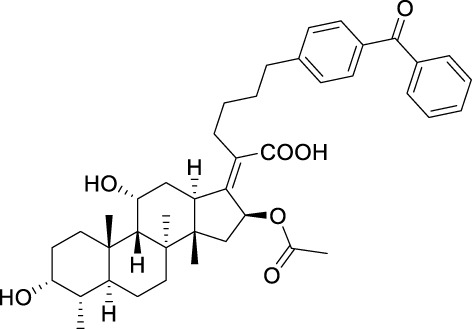	1–4 μg/mL (Riber et al., 2006)	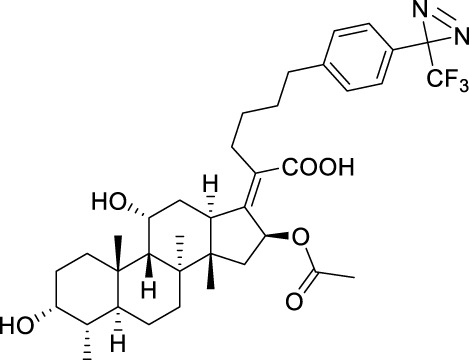	1–4 μg/mL (Riber et al., 2006)
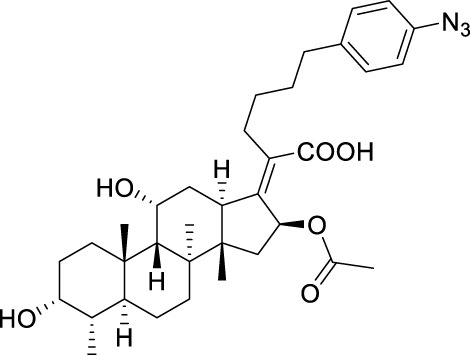	1–4 μg/mL (Riber et al., 2006)	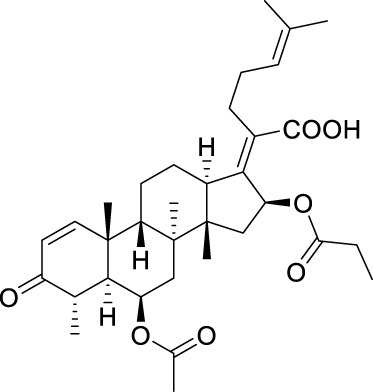	16 μg/mL (Kong et al., 2018)
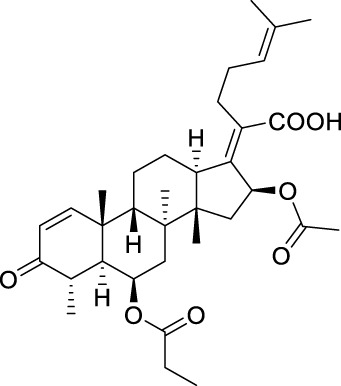	2 μg/mL (Kong et al., 2018)	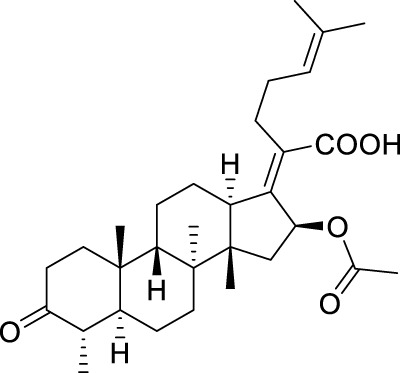	2 μg/mL (Lv et al., 2017)
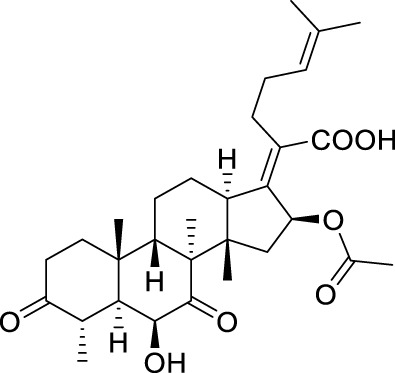	1 μg/mL (Lv et al., 2017)	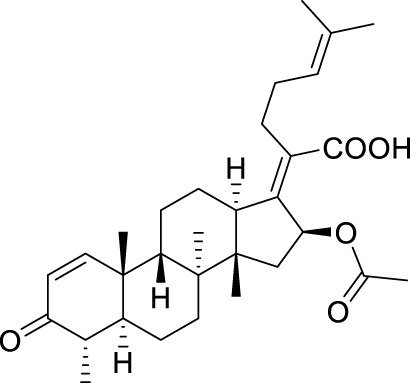	1 μg/mL (Lv et al., 2017)
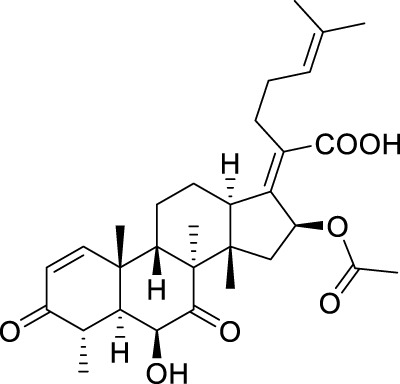	0.5 μg/mL (Lv et al., 2017)	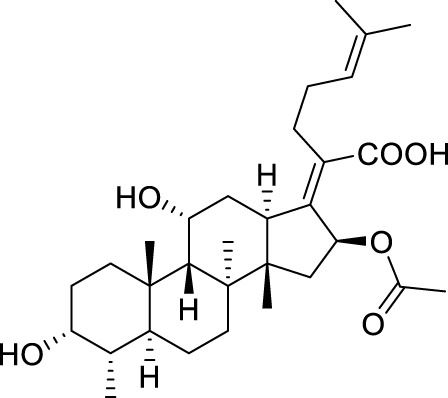	0.125 μg/mL (Garcia Chavez et al., 2021)
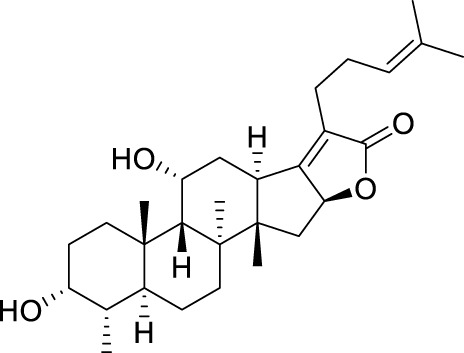	4.0μg/mL (Godtfredsen et al., 1966	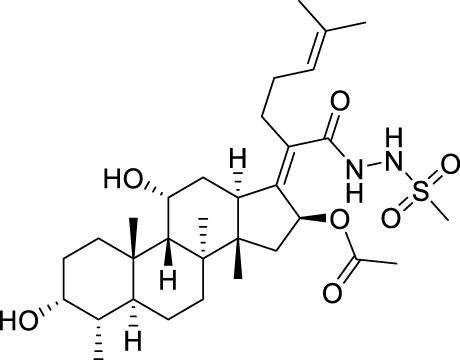	10 μM (Singh et al., 2020)
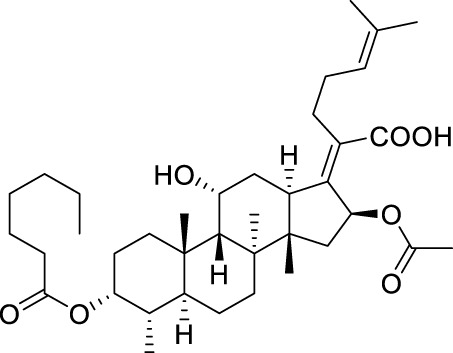	5 μM (Singh et al., 2020)	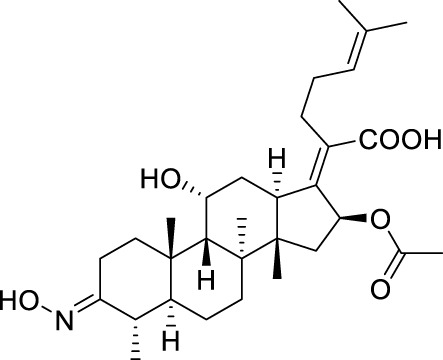	2.5 μM (Singh et al., 2020)
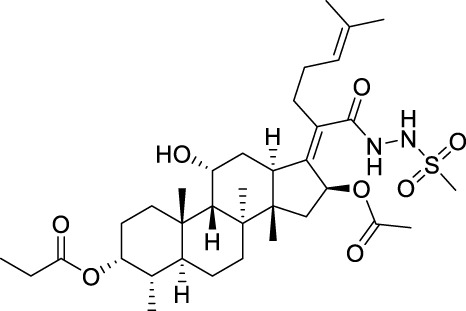	7.81 μM (Singh et al., 2020)		

### 3.2 Molecular docking

To anticipate the ligand–receptor interactions, molecular docking experiments were conducted using SYBYL-X 2.0 software. The target protein EF-G (PDB: 2XEX) of *S. aureus* was selected as a receptor to bind the derivatives, and several significant residues were identified as the active protein pocket. The **FA**-binding site was bordered by Arg464, His457, Leu456, Thr436, Asp434, and Phe88, which created a specific cavity. All the hydrogens were added to EF-G in the structural model to improve the quality of the model. The Tripos force field and Gasteiger–Huckel charges were assigned for the EF-G and **FA** derivatives, respectively. After docking, the ligand–receptor complexes were opened in PyMol software for the visualizer to analyze the interaction.

### 3.3 Materials

All reagents were purchased from commercial suppliers of Adamas Reagent Ltd. (Shanghai, China) in analytical reagent grade and were used directly without further purification. Flash chromatography was carried out with silica gel (200–300 mesh) which was supplied by Innochem Co., Ltd. (Beijing, China). Analytical TLC was performed on pre-coated silica gel F254 plates (0.25 mm; E. Merck), and the products were visualized by UV detection or treated with an ethanolic solution of p-anisaldehyde spray followed by heating. The derivatives of **FA** were characterized by ^1^H NMR, ^13^C NMR, HRMS, and elemental analysis. The antibacterial activity was assayed by using a multi-model plate reader (Infinite 200).

### 3.4 Synthesis chemistry

#### 3.4.1 21-Fusidic acid (pivaloyloxymethyl) ester (FA-1, C_37_H_58_O_8_)

First, chloromethyl pivalate (1.52 ml; 9.062 mmol) was added to the solution of **FA** (2 g; 4.513 mmol) in dry N, N-dimethylformamide (30 ml) at room temperature for 10 min. This was followed by drop-wise addition of triethylamine (0.7 mL; 5.890 mmol). The resulting reaction mixture was stirred at 50°C overnight. After completion of the reaction (TLC), the mixture was diluted with EtOAc and washed with water. The EtOAc layer was then dried over anhydrous sodium sulfate, filtered, and concentrated *in vacuo*. The crude product was purified by column chromatography using n-hexane: ethyl acetate = 1: 4 as the eluent, affording the target compound as a white solid (1.62 g; 81%). Mp: 76°C–78°C. ^1^H NMR (400 MHz, CDCl_3_) δ 7.27 (s, 1H), 5.86 (d, J = 8.3 Hz, and 1H), 5.74 (dd, 2H), 5.08 (t, J = 7.0 Hz, and 1H), 4.34 (s, 1H), 3.74 (d, J = 8.3 Hz, and 1H), 3.05 (d, J = 11.1 Hz, and 1H), 2.54–2.36 (m, 2H), 2.31 (d, J = 14.1 Hz, and 1H), 2.25–2.06 (m, 4H), 1.98 (s, 3H), 1.93–1.79 (m, 2H), 1.79–1.70 (m, 2H), 1.67 (s, 3H), 1.61–1.47 (m, 8H), 1.37 (s, 3H), 1.29 (t, J = 16.0, 7.6 Hz, and 2H), 1.21 (s, 9H), 1.18–1.02 (m, 2H), 0.97 (s, 3H), 0.90 (d, J = 16.7, 7.5 Hz, and 6H). ^13^C NMR (100 MHz, CDCl_3_) δ 176.0, 171.3, 169.1, 151.9, 131.6, 128.3, 123.9, 79.8, 74.3, 71.4, 68.2, 49.2, 48.8, 44.3, 39.4, 39.0, 38.8, 37.1, 36.2, 36.2, 35.6, 32.4, 30.3, 30.0, 28.8, 28.2, 26.9, 25.7, 24.2, 22.8, 20.8, 20.8, 17.9, 17.8, 15.9. Anal. calcd. for C_37_H_58_O_8_: C 70.44, H 9.27; found: C 69.81, H 9.32. HRMS (ESI): C_37_H_58_O_8_Na (653.4029) [M + Na]^+^ = 653.4027.

#### 3.4.2 24-Oxo-21-fusidic acid (pivaloyloxymethyl) ester (FA-2, C_34_H_52_O_9_)

In a 50-mL round-bottom flask, **FA-1** (100 mg; 0.166 mmol) was dissolved in dichloromethane (5 mL). Then NMO (18.56 mg; 0.249 mmol) and OsO_4_ (0.9 μL, 0.0166 mmol, and 0.1 eq.) in MeCN were added, respectively. The solution of 2% O_3_/O_2_ (nominal output of 1 mmol O_3_/min) was introduced directly above the solution *via* a glass pipet for 6.6 min (nominally 2.2 equiv ozone relative to alkene) at 0°C for 1 h. The reaction was then quenched by the addition of 10 mL of saturated sodium thiosulfate. The reaction was stirred for an additional 45 min and then concentrated *in vacuo* to remove dichloromethane. Subsequently, EtOAc and brine were added, and the layers were separated. The aqueous layer was extracted with EtOAc. The combined organic layers were dried over anhydrous sodium sulfate, filtered, and concentrated *in vacuo*. The crude product was purified by flash chromatography using an eluent (n-hexane: ethyl acetate = 3: 2, V: V). **FA-2** as a white solid was obtained. Yield: 90%. ^1^H NMR (400 MHz, CDCl_3_) δ 9.76 (s, 1H), 5.96 (d, J = 8.4 Hz, 1H), 5.74 (dd, J = 28.0, 5.4 Hz, 2H), 4.35 (s, 1H), 3.71 (d, J = 2.0 Hz, 1H), 3.10 (d, J = 10.6 Hz, 1H), 2.75–2.46 (m, 5H), 2.39–2.24 (m, 2H), 2.18 (d, J = 3.9 Hz, 2H), 1.98 (s, 3H), 1.90–1.40 (m, 8H), 1.36 (s, 3H), 1.28 (dd, J = 12.2, 11.0 Hz, 2H), 1.21 (s, 9H), 1.17–1.03 (m, 2H), 0.97 (s, 3H), 0.90 (d, J = 6.4 Hz, 6H). ^13^C NMR (100 MHz, CDCl_3_) δ 201.2, 177.1, 170.1, 167.4, 153.2, 127.1, 79.9, 74.2, 71.4, 67.9, 49.4, 48.8, 44.6, 43.8, 39.5, 38.8, 38.7, 36.8, 36.6, 35.5, 35.3, 31.3, 30.9, 29.8, 26.8, 23.6, 23.4, 21.3, 21.1, 20.8, 17.7, and 16.0. HRMS (ESI): C_34_H_52_O_9_Na (627.3509) [M + Na]^+^ = 627.3503.

#### 3.4.3 24-Ene-21-fusidic acid (pivaloyloxymethyl) ester (FA-3, C_35_H_54_O_8_)

A solution of methyltriphenylphosphonium bromide (389.04 mg; 1.09 mmol) and potassium tert-butoxide (122.2 mg, 1.09 mol) was added to the mixture of **FA-2** (441.49 mg, 0.73 mol) in 20 mL of toluene under nitrogen. The reaction was kept refluxed overnight and monitored by TLC. After completion of the reaction (TLC), the mixture was diluted with EtOAc and washed with water. The EtOAc layer was then dried over anhydrous sodium sulfate, filtered, and concentrated *in vacuo*. The crude product was purified by flash chromatography using an eluent (n-hexane: ethyl acetate = 3: 2, V: V). **FA-3** as a white solid was obtained. Yield: 75%. Anal. calcd. for C_35_H_54_O_8_: C 69.74, H 9.03; found: C 68.93, H 8.96. HRMS (ESI): C_35_H_54_O_8_Na (625.3716) [M + Na]^+^ = 625.3709 (Zhao et al., 2016).

#### 3.4.4 (E)-25-chlorohexa-24-ene-21-fusidic acid (pivaloyloxymethyl)ester (FA-4, C_35_H_53_ClO_8_)

A solution of **FA-2** (200 mg, 0.34 mmol) and (chloromethyl) triphenylphosphonium chloride (212 mg, 0.68 mmol) in anhydrous tetrahydrofuran (15 mL) was cooled to 0°C under nitrogen for 15 min, and then n-butyllithium (416.7 μL) dissolved in n-hexane (0.68 mmol, 1.6 mol/L) was added drop-wise above the solution and stirred at 0°C for 30 min. After completion of the reaction (TLC), the mixture was diluted with EtOAc and washed with water. The EtOAc layer was then dried over anhydrous sodium sulfate, filtered, and concentrated *in vacuo*. The crude product was purified by flash chromatography using an eluent (n-hexane: ethyl acetate = 3: 2, V: V). **FA-4** as a white solid was obtained. Yield: 43% (Zhao et al., 2016).

#### 3.4.5 (E)-25-bromohexa-24-ene-21-fusidic acid (pivaloyloxymethyl)ester (FA-5, C_35_H_53_BrO_8_)

A solution of **FA-2** (200 mg, 0.34 mmol) and (bromomethyl) triphenylphosphonium bromide (296.7 mg, 0.68 mmol) in anhydrous tetrahydrofuran (15 mL) was cooled to 0°C under nitrogen for 15 min, and then, n-butyllithium (416.7 μL) dissolved in n-hexane (0.68 mmol, 1.6 mol/L) was added drop-wise above the solution and stirred at 0°C for 30 min. After completion of the reaction (TLC), the mixture was diluted with EtOAc and washed with water. The EtOAc layer was then dried over anhydrous sodium sulfate, filtered, and concentrated *in vacuo*. The crude product was purified by flash chromatography using an eluent (n-hexane: ethyl acetate = 3: 2, V: V). **FA-5** as a white solid was obtained. Yield: 32%. ^1^H NMR (400 MHz, CDCl_3_) δ 6.22–6.11 (m, 1H), 6.11–6.00 (m, 1H), 5.93–5.84 (m, 1H), 5.80 (dd, J = 8.9, 5.4 Hz, 1H), 5.70 (d, J = 5.4 Hz, 1H), 4.34 (s, 1H), 3.75 (s, 1H), 3.07 (t, J = 12.2 Hz, 1H), 2.64–2.44 (m, 2H), 2.42–2.05 (m, 6H), 1.98 (d, J = 1.6 Hz, 3H), 1.93–1.69 (m, 4H), 1.65–1.48 (m, 4H), 1.37 (s, 3H), 1.35–1.23 (m, 3H), 1.21 (s, 9H), 1.27–1.03 (m, 2H), 0.99 (s, 3H), and 0.92 (d, J = 6.4 Hz, 6H). ^13^C NMR (100 MHz, CDCl_3_) δ 177.1, 170.2, 167.7, 152.5, 136.4, 133.2, 128.2, 108.7, 105.5, 79.8, 74.3, 71.4, 68.2, 49.2, 48.9, 44.5, 39.5, 39.0, 38.8, 37.1, 36.2, 36.2, 35.7, 32.5, 30.3, 30.0, 29.8, 27.0, 26.9, 24.2, 22.7, 20.8, 20.7, 18.0, and 15.9. HRMS (ESI): C_35_H_53_BrNaO_8_ (703.2822) [M + Na]^+^ = 703.2606.

#### 3.4.6 General procedures to produce FA-6, FA-7, and FA-8

A solution of derivatives (**FA-3**, **FA-4,** and **FA-5**, respectively; 0.0924 mmol) and potassium carbonate (25.55 mg, 0.185 mmol) in methanol was stirred at room temperature for 1 h and monitored by TLC. After completion of the reaction, the mixture was diluted with EtOAc and washed with water. The EtOAc layer was then dried over anhydrous sodium sulfate, filtered, and concentrated *in vacuo*. The crude product was purified by flash chromatography using an eluent (n-hexane: ethyl acetate = 3: 2, V: V). **FA-6**, **FA-7**, and **FA-8** were obtained, respectively.

24-ene-Fusidic acid (**FA-6**). White solid. Yield: 55%. ^1^H NMR (400 MHz, CDCl_3_) δ 5.88 (d, J = 8.3 Hz, 1H), 5.86–5.73 (m, 1H), 5.04 (d, J = 17.1 Hz, 1H), 4.97 (d, J = 10.1 Hz, 1H), 4.34 (s, 1H), 3.73 (s, 1H), 3.05 (d, J = 12.0 Hz, 1H), 2.45–2.41 (m, 2H), 2.29 (d, J = 13.8 Hz, 1H), 2.24–2.03 (m, 5H), 1.99 (s, 3H), 1.84 (t, J = 13.2 Hz, 2H), 1.78–1.66 (m, 2H), 1.66–1.53 (m, 3H), 1.49 (d, J = 12.8 Hz, 1H), 1.43 (s, 1H), 1.38 (s, 3H), 1.29 (d, J = 5.6 Hz, 1H), 1.26 (s, 1H), 1.18–1.05 (m, 2H), 0.98 (s, 3H), and 0.92 (s, 6H). ^13^C NMR (100 MHz, CDCl_3_) δ 172.4, 171.4, 148.6, 137.6, 130.1, 115.1, 74.4, 71.4, 68.2, 49.4, 48.8, 43.9, 39.6, 38.9, 36.8, 36.6, 35.8, 35.5, 33.8, 31.8, 30.0, 29.8, 28.0, 23.6, 23.3, 21.1, 20.6, 17.6, and 15.9. Anal. calcd. for C_29_H_44_O_6_: C 71.28, H 9.08; found: C 70.42, H 9.12. HRMS (TOF): C_29_H_43_O_6_ (487.3060) [M-H]^-^ = 487.3058.

(E)-25-chlorohexa-24-ene-fusidic acid (**FA-7**). White solid. Yield: 55%. ^1^H NMR (400 MHz, CDCl_3_) δ 6.02 (dd, J = 19.0, 10.2 Hz, 1H), 5.95–5.70 (m, 2H), 4.36 (s, 1H), 3.76 (s, 1H), 3.08 (t, J = 10.3 Hz, 1H), 2.65–2.48 (m, 2H), 2.48–2.21 (m, 3H), 2.21–2.06 (m, 3H), 1.97 (s, 3H), 1.92–1.67 (m, 4H), 1.67–1.46 (m, 4H), 1.38 (s, 3H), 1.31 (d, J = 14.3 Hz, 1H), 1.28–1.18 (m, 2H), 1.18–1.03 (m, 2H), 0.98 (s, 3H), and 0.92 (d, J = 5.4 Hz, 6H). ^13^C NMR (100 MHz, CDCl_3_) δ 173.9 [173.7 (for the second diastereoisomer)], 170.7, 152.44 [152.39 (for the second diastereoisomer)], 132.5 [130.2 (for the second diastereoisomer)], 128.6 [128.3 (for the second diastereoisomer)], 118.9 [118.0 (for the second diastereoisomer)], 74.44 [74.40 (for the second diastereoisomer)], 71.6, 68.2 [68.1 (for the second diastereoisomer)], 49.27 [49.26 (for the second diastereoisomer)], 48.78 [48.77 (for the second diastereoisomer)], 44.50 [44.45 (for the second diastereoisomer)], 39.5, 38.90 [38.88 (for the second diastereoisomer)], 36.9, 36.4, 36.0 [35.9 (for the second diastereoisomer)], 35.6 [35.5 (for the second diastereoisomer)], 32.08 [32.06 (for the second diastereoisomer)], 31.2 [27.9 (for the second diastereoisomer)], 30.3 [30.1 (for the second diastereoisomer)], 29.8 [27.4 (for the second diastereoisomer)], 27.2 [27.0 (for the second diastereoisomer)], 24.0, 23.06 [23.03 (for the second diastereoisomer)], 20.9, 20.6, 17.9, and 16.0. Anal. calcd. for C_29_H_43_ClO_6_: C 66.59, H 8.29; found: C 65.21, H 8.34. HRMS (ESI): C_29_H_42_ClO_6_ (521.2670) [M-H]^-^ = 521.2656.

(E)-25-bromohexa-24-ene-fusidic acid (**FA-8**). White solid. Yield: 80%. ^1^H NMR (400 MHz, CDCl_3_) δ 6.22–6.15 (m, 1H), 6.15–6.03 (m, 1H), 5.92 (d, J = 8.3 Hz, 1H), 4.35 (s, 1H), 3.76 (s, 1H), 3.08 (t, J = 12.0 Hz, 1H), 2.62–2.46 (m, 2H), 2.46–2.06 (m, 6H), 1.98 (s, 3H), 1.93–1.67 (m, 4H), 1.67–1.46 (m, 4H), 1.38 (s, 3H), 1.35–1.23 (m, 2H), 1.21 (t, J = 7.0 Hz, 1H), 1.18–1.03 (m, 2H), 0.98 (s, 3H), and 0.95–0.88 (m, 6H). ^13^C NMR (100 MHz, CDCl_3_) δ 173.7, 170.9, 152.3, 136.8 [133.6 (for the second diastereoisomer)], 128.9, 108.7 [105.6 (for the second diastereoisomer)], 74.5, 71.7, 68.4, 49.4, 49.0, 44.6, 39.6, 39.1, 37.1, 36.4, 36.2, 35.8, 33.2, 32.4 [30.3 (for the second diastereoisomer)], 30.2, 30.0, 27.2, 24.2, 23.0, 21.0, 20.8, 18.1, and 16.1. Anal. calcd. for C_29_H_43_BrO_6_: C 61.37, H 7.64; found: C 60.41, H 7.66. HRMS (ESI): C_29_H_42_^79^BrO_6_ (565.2165) [M-H]^-^ = 565.2147; C_29_H_42_^81^BrO_6_ (567.2144) [M-H]^-^ = 567.2146.

#### 3.4.7 Fusidic acid [b]furan-21-one (FA-9, C_29_H_44_O_4_)

A solution of **FA** (200 mg, 0.32 mmol) and sodium hydroxide (1.95 mmol, 1.2 mL) in methanol was refluxed overnight and monitored by TLC. After completion of the reaction, 1 N hydrochloric acid was added to adjust the pH to 2–3. The mixture was diluted with EtOAc and washed with water. The EtOAc layer was then dried over anhydrous sodium sulfate, filtered, and concentrated *in vacuo*. The crude product was purified by flash chromatography using an eluent (n-hexane: ethyl acetate = 1: 1, V: V). **FA-9** as a white solid was obtained. Yield: 74%. ^1^H NMR (400 MHz, CDCl_3_) δ 5.11 (t, J = 6.0 Hz, 1H), 4.95 (dd, J = 10.9, 4.2 Hz, 1H), 4.39 (s, 1H), 3.75 (s, 1H), 3.53 (d, J = 11.7 Hz, 1H), 2.41–2.15 (m, 6H), 2.15–1.96 (m, 4H), 1.90–1.78 (m, 2H), 1.75 (d, J = 13.3 Hz, 1H), 1.68 (s, 3H), 1.66–1.62 (m, 1H), 1.60 (s, 3H), 1.57–1.46 (m, 6H), 1.32–1.07 (m, 4H), 0.97 (s, 3H), 0.94 (d, J = 6.7 Hz, 3H), and 0.82 (s, 3H). ^13^C NMR (100 MHz, CDCl_3_) δ 176.8, 169.1, 132.9, 123.6, 123.4, 82.0, 71.5, 68.0, 55.3, 50.6, 40.9, 38.3, 37.2, 36.9, 36.0, 34.2, 31.8, 31.6, 30.2, 30.0, 27.6, 25.8, 24.2, 23.5, 23.2, 21.2, 20.1, 17.9, and 16.1. Anal. calcd. for C_29_H_44_O_4_: C 76.27, H 9.71; found: C 74.91, H 9.57. HRMS (TOF): C_29_H_44_O_4_Na (479.3137) [M + Na]^+^ = 479.3136.

#### 3.4.8 3β-(Methylsulfonyloxy)-21-fusidic acid (pivaloyloxymethyl) ester (FA-10, C_38_H_60_O_10_S)

A solution of **FA-1** (615 mg, 0.98 mmol) and pyridine (236.8 μL, 2.4 mmol) in anhydrous dichloromethane (20 mL) was stirred at 0°C for 15 min and methane sulfonyl chloride (1.95 mmol, 151.23 μL) was added drop-wise. The reaction was stirred overnight and monitored by TLC. After completion of the reaction, 1 N hydrochloric acid was added to adjust pH to 2–3. The mixture was diluted with EtOAc and washed with water. The EtOAc layer was then dried over anhydrous sodium sulfate, filtered, and concentrated *in vacuo*. The crude product was purified by flash chromatography. **FA-10** as a white solid was obtained. Yield: 60%. HRMS (TOF): C_38_H_60_O_10_NaS (731.3805) [M + Na]^+^ = 731.3812.

#### 3.4.9 3α-Chloro-21-fusidic acid (pivaloyloxymethyl) ester (FA-11, C_37_H_57_ClO_7_)

A solution of **FA-10** (100 mg, 0.141 mmol) and tetrabutylammonium chloride (78.5 mg, 0.282 mmol) in tetrahydrofuran (5 mL) was stirred at 80°C for 1 h and monitored by TLC. After completion of the reaction, the mixture was diluted with EtOAc and washed with water. The EtOAc layer was then dried over anhydrous sodium sulfate, filtered, and concentrated *in vacuo*. The crude product was purified by flash chromatography. **FA-11** as a white solid was obtained. Yield: 49.1%. HRMS (TOF): C_37_H_57_O_7_NaCl (671.3691) [M + Na]^+^ = 671.3678.

#### 3.4.10 3α-Bromo-21-fusidic acid (pivaloyloxymethyl) ester (FA-12, C_37_H_57_BrO_7_)

A solution of **FA-10** (100 mg, 0.141 mmol) and tetrabutylammonium bromine (91.0 mg, 0.282 mmol) in dimethyl sulfoxide (5 mL) was stirred at room temperature overnight and monitored by TLC. After completion of the reaction, the mixture was diluted with EtOAc and washed with water. The EtOAc layer was then dried over anhydrous sodium sulfate, filtered, and concentrated *in vacuo*. The crude product was purified by flash chromatography. **FA-12** as a white solid was obtained. Yield: 51.0%. HRMS (ESI): C_37_H_57_O_7_Na^79^Br (715.3171) [M + Na]^+^ = 715.3185; C_37_H_57_O_7_Na^81^Br (717.3177) [M + Na]^+^ = 717.3165.

#### 3.4.11 3α-Iodo-21-fusidic acid (pivaloyloxymethyl) ester (FA-13, C_37_H_57_IO_7_)

A solution of **FA-10** (100 mg, 0.141 mmol) and tetrabutylammonium iodide (104.3 mg, 0.282 mmol) in tetrahydrofuran (5 mL) was stirred at room temperature for 6 h and monitored by TLC. After completion of the reaction, the mixture was diluted with EtOAc and washed with water. The EtOAc layer was then dried over anhydrous sodium sulfate, filtered, and concentrated *in vacuo*. The crude product was purified by flash chromatography. **FA-13** as a white solid was obtained. Yield: 40.5%. HRMS (ESI): C_37_H_57_O_7_NaI (763.3047) [M + Na]^+^ = 763.3053.

#### 3.4.12 3α-Azido-21-fusidic acid (pivaloyloxymethyl) ester (FA-14, C_37_H_57_N_3_O_7_)

A solution of **FA-10** (100 mg, 0.141 mmol) and sodium azide (18.3 mg, 0.282 mmol) in dimethyl sulfoxide (5 mL) was stirred at 90°C overnight and monitored by TLC. After completion of the reaction, the mixture was diluted with EtOAc and washed with water. The EtOAc layer was then dried over anhydrous sodium sulfate, filtered, and concentrated *in vacuo*. The crude product was purified by flash chromatography. **FA-14** as a white solid was obtained. Yield: 15.7%. HRMS (ESI): C_37_H_57_N_3_O_7_Na (678.4094) [M + Na]^+^ = 678.4101.

#### 3.4.13 3α-Nitrohexadecahydro-21-fusidic acid (pivaloyloxymethyl) ester (FA-15, C_37_H_57_NO_9_)

A solution of **FA-10** (100 mg, 0.141 mmol) and tetrabutylammonium nitrate (85.9 mg, 0.282 mmol) in dimethyl sulfoxide (5 mL) was stirred at 70°C overnight and monitored by TLC. After completion of the reaction, the mixture was diluted with EtOAc and washed with water. The EtOAc layer was then dried over anhydrous sodium sulfate, filtered, and concentrated *in vacuo*. The crude product was purified by flash chromatography. **FA-15** as a white solid was obtained. HRMS (ESI): C_37_H_57_NO_9_Na (682.3931) [M + Na]^+^ = 682.3917.

#### 3.4.14 3α-Phenylamino-21-fusidic acid (pivaloyloxymethyl) ester (FA-16, C_43_H_63_NO_7_)

A solution of **FA-10** (100 mg, 0.141 mmol), triethylamine (28.6 mg, 0.282 mmol), and aniline (26.3 mg, 0.282 mmol) in tetrahydrofuran (5 mL) was stirred at 90°C overnight and monitored by TLC. After completion of the reaction, the mixture was diluted with EtOAc and washed with water. The EtOAc layer was then dried over anhydrous sodium sulfate, filtered, and concentrated *in vacuo*. The crude product was purified by flash chromatography. **FA-16** as a white solid was obtained. Yield: 45.2%. HRMS (ESI): C_43_H_64_NO_7_ (706.4683) [M + H]^+^ = 706.4667.

#### 3.4.15 3-Ene-21-fusidic acid (pivaloyloxymethyl) ester (FA-23, C_37_H_56_O_7_)

A solution of **FA-10** (0.759 g, 1.32 mmol, 1.0 equiv) in 2,6-lutidine (5.00 mL) was heated to 130°C and stirred at the same temperature for 2 h and monitored by TLC. Upon completion, the reaction mixture was cooled to 23°C and then concentrated directly. A white solid was obtained. Yield: 95.5%. HRMS (ESI): C_37_H_56_O_7_Na (635.3924) [M + Na]^+^ = 635.3918.

#### 3.4.16 General procedures to produce FA-17∼FA-22 and FA-24

A solution of the derivatives (**FA-11**∼**FA-16** and **FA-23**, respectively; 0.185 mmol) and potassium carbonate (25.55 mg, 0.185 mmol) in methanol (5 mL) was stirred at room temperature for 1 h and monitored by TLC. After completion of the reaction, the mixture was diluted with EtOAc and washed with water. The EtOAc layer was then dried over anhydrous sodium sulfate, filtered, and concentrated *in vacuo*. The crude product was purified by flash chromatography using an eluent (n-hexane: ethyl acetate = 1: 1, V: V). The derivatives (**FA-17∼24**) were obtained, respectively.

3α-Chloro-21-fusidic acid (**FA-17**, C_31_H_47_O_5_Cl). White solid, Yield: 97%. HRMS (ESI): C_31_H_47_O_5_NaCl (557.3010) [M + Na]^+^ = 557.2997.
3α-Bromo-21-fusidic acid (**FA-18**, C_31_H_47_O_5_Br). White solid. Yield: 85.5%. HRMS (ESI): C_31_H_46_O_5_^79^Br (577.2529) [M-H]^-^ = 577.2534; C_31_H_46_O_5_^81^Br (579.2508) [M-H]^-^ = 579.2522.
3α-Iodo-21-fusidic acid (**FA-19**, C_31_H_47_O_5_I). White solid. Yield: 97.6%. HRMS (ESI): C_31_H_46_O_5_I (625.2390) [M-H]^-^ = 625.2380.
3α-Azido-21-fusidic acid (**FA-20**, C_31_H_47_N_3_O_5_). White solid. Yield: 58.0%. HRMS (ESI): C_31_H_46_N_3_O_5_ (540.3437) [M-H]^-^ = 540.3428.
3α-Nitrohexadecahydro-21-fusidic acid (**FA-21**, C_31_H_48_NO_7_). White solid. Yield: 58.0%.
3α-Phenylamino-21-fusidic acid **(FA-22**, C_37_H_53_NO_5_). White solid. Yield: 65.5%. HRMS (ESI): C_37_H_52_NO_5_ (590.3845) [M-H]^-^ = 590.3838.
3-Ene-21-fusidic acid (**FA-24**, C_31_H_46_O_5_). White solid. Yield: 98.3%. HRMS (ESI): C_31_H_46_O_5_Na (521.3243) [M + Na]^+^ = 521.3235.

### 3.5 Biological evaluation

#### 3.5.1 Inhibition zone test

The standard agar diffusion method with a slight modification was used for the determination of the antibacterial efficacy of the **FA** derivatives (Luangtongkum et al., 2007; Gaudreau et al., 2008; Benamrouche et al., 2014). *S. aureus* (ATCC 6538), S. albus (ATCC 29213), S. epidermidis (ATCC 12228), *S. typhimurium* (CMCC 50115), and *E. coli* (CMCC 44102) were cultured in a liquid medium, Mueller–Hinton Agar (MHA), at 37°C. Bacterial suspensions of 1.5 × 10^6^ CFU/mL with 400 μL prepared were uniformly inoculated onto MHA solidified in 60-mm Petri dishes. Sterile filter paper disks of 6 mm diameter containing 5 μL different concentrations of compounds were pressed gently against the surface of the agar. A disk containing Gatifloxacin was used as a positive control, while DMSO was used as the negative control. Then the disks were incubated in a constant temperature incubator at 37°C for 24 h and the bacteriostatic circles were observed. The inhibition zone (IZ) diameter was measured using a vernier caliper. All the experiments were performed in triplicate.

#### 3.5.2 Minimum inhibitory concentration (MIC) assay

The MIC was determined by a microdilution method in 96-well plates according to the Clinical and Laboratory Standards Institute (CLSI) with a slight modification. (Sader et al., 2006). Liquid media were used to cultivate the test bacteria at 37°C. Then, 195-μL bacterial suspensions containing 1.5 × 10^5^ CFU/mL with 5 μL different derivative concentrations were added to 96-cell plates and the plates were incubated at 37°C for 24 h. In each well, OD values of derivatives were measured at 600 nm and compared with blank controls without bacteria and negative controls with bacteria. The lowest concentration of compounds, which did not show any visible growth of the test organisms after macroscopic evaluation, was determined as the MIC. Gatifloxacin served as the positive control and DMSO served as the negative control.

#### 3.5.3 Quantitative structure–activity relationship (QSAR) study

The MIC values (μM) of the constructed 22 derivatives (**FA**, **FA-1**, **FA-2**, and **FA-6**∼**24)** were converted into their corresponding negative logarithms (pMIC) for the 3D-QSAR model analysis by SYBYL-X 2.0 software (Shanghai Tri-I. Biotech. Inc., China) (Wang et al., 2019; Chen et al., 2020). Three-dimensional molecular conformations were successively optimized using the Gasteiger−Huckel charge, Tripos force field, and Powell conjugate gradient algorithm until the obtained convergence criteria were minimized in molecular energies. Three-dimensional structures of derivatives were aligned on the common scaffold of the template molecule **FA-7** that exhibited the best *in vitro* antibacterial activity against Gram-positive bacteria among the 22 synthesized derivatives. A partial least-squares (PLS) technique was applied for optimizing the obtained 3D-QSAR model. Subsequently, the obtained PLS coefficients and standard descriptor values were used to generate their corresponding contour maps including steric, electrostatic, hydrophobic, and hydrogen bond acceptors.

## 4 Conclusion

In this study, a ligand-based pharmacophore model was constructed and seven **FA** derivatives were designed according to the reported structure–activity relationship and the pharmacophore characteristics. The designed **FA** derivatives were applied to analyze the matching degree with the pharmacophore model through Qfit values, and partially designed **FA** derivatives were docked onto the EF-G of *S. aureus* to study the bonding with the target protein. Finally, the designed **FA** derivatives were synthesized and their antibacterial activities were evaluated by the inhibition zone test and the MIC test. Afterward, 3D-QSAR was carried out on all the derivatives, and the results indicated that the substituents at the C-3, C-21, and C-25 positions would exert an influence on the antibacterial activity of derivatives. In summary, this study provides a promising computational approach to design **FA** derivatives with highly potent antibacterial activity.

## Data Availability

The original contributions presented in the study are included in the article/Supplementary Material; further inquiries can be directed to the corresponding authors.

## References

[B1] Belardinelli R. Rodnina M. V. (2017). Effect of fusidic acid on the kinetics of molecular motions during EF-G-induced translocation on the ribosome. Sci. Rep. 7, 10536. 10.1038/s41598-017-10916-8 28874811 PMC5585275

[B2] Benamrouche N. Lazri M. Hassiba T. M. Rahal K. (2014). Comparison of Corynebacterium diphtheriae susceptibility testing to antibiotics by the broth dilution and diffusion (E-test and disk) methods. Med. Maladies Infect. 44, 392–393. 10.1016/j.medmal.2014.07.007 25149268

[B3] Bodley J. W. Zieve F. J. Lin L. Zieve S. T. (1969). Formation of the ribosome-G factor-GDP complex in the presence of fusidic acid. Biochem. Biophysical Res. Commun. 37, 437–443. 10.1016/0006-291X(69)90934-6 4900137

[B4] Borg A. Holm M. Shiroyama I. Hauryliuk V. Pavlov M. Sanyal S. (2015). Fusidic acid targets elongation factor G in several stages of translocation on the bacterial ribosome. J. Biol. Chem. 290, 3440–3454. 10.1074/jbc.M114.611608 25451927 PMC4319013

[B5] Cardoso M. H. Orozco R. Q. Rezende S. B. Rodrigues G. Oshiro K. G. N. Candido E. S. (2019). Computer-aided design of antimicrobial peptides: Are we generating effective drug candidates? Front. Microbiol. 10, 3097. 10.3389/fmicb.2019.03097 32038544 PMC6987251

[B6] Cerqueira N. M. F. S. A. Gesto D. Oliveira E. F. Santos-Martins D. Brás N. F. Sousa S. F. (2015). Receptor-based virtual screening protocol for drug discovery. Archives Biochem. Biophysics 582, 56–67. 10.1016/j.abb.2015.05.011 26045247

[B7] Chen J. Luo Y. Wei C. Wu S. Wu R. Wang S. (2020). Novel sulfone derivatives containing a 1, 3, 4-oxadiazole moiety: Design and synthesis based on the 3D-QSAR model as potential antibacterial agent. Pest Manag. Sci. 76, 3188–3198. 10.1002/ps.5873 32343024

[B8] Chen Y. Koripella R. K. Sanyal S. Selmer M. (2010). Staphylococcus aureus elongation factor G – structure and analysis of a target for fusidic acid. FEBS J. 277, 3789–3803. 10.1111/j.1742-4658.2010.07780.x 20718859

[B9] Chen Z. Li H. Zhang Q. Bao X. Yu K. Luo X. (2009). Pharmacophore-based virtual screening versus docking-based virtual screening: A benchmark comparison against eight targets. Acta Pharmacol. Sin. 30, 1694–1708. 10.1038/aps.2009.159 19935678 PMC4007494

[B10] Collignon P. Turnidge J. (1999). Fusidic acid *in vitro* activity. Int. J. Antimicrob. Agents 12, S45–S58. 10.1016/S0924-8579(98)00073-9 10528786

[B11] Duvold T. Sørensen M. D. Björkling F. Henriksen A. S. Rastrup-Andersen N. (2001). Synthesis and conformational analysis of fusidic acid side chain derivatives in relation to antibacterial activity. J. Med. Chem. 44, 3125–3131. 10.1021/jm010899a 11543681

[B12] Fjell C. D. Hiss J. A. Hancock R. E. W. Schneider G. (2012). Designing antimicrobial peptides: Form follows function. Nat. Rev. Drug Discov. 11, 37–51. 10.1038/nrd3591 22173434

[B13] Garcia Chavez M. Garcia A. Lee H. Y. Lau G. W. Parker E. N. Komnick K. E. (2021). Synthesis of fusidic acid derivatives yields a potent antibiotic with an improved resistance profile. ACS Infect. Dis. 7, 493–505. 10.1021/acsinfecdis.0c00869 33522241 PMC8713577

[B14] Gaudreau C. Girouard Y. Gilbert H. Gagnon J. Bekal S. (2008). Comparison of disk diffusion and agar dilution methods for erythromycin, ciprofloxacin, and tetracycline susceptibility testing of campylobacter coli and for tetracycline susceptibility testing of campylobacter jejuni subsp jejuni. Antimicrob. Agents Chemother. 52, 4475–4477. 10.1128/AAC.00767-08 18838597 PMC2592876

[B15] Godtfredsen W. O. Albrethsen C. Daehne W. V. Tybring L. Vangedal S. (1965). Transformations of fusidic acid and the relationship between structure and antibacterial activity. Antimicrob. Agents Chemother. (Bethesda) 5, 132–137. 10.1021/jm00319a004 5883417

[B16] Godtfredsen W. O. Von Daehne W. Tybring L. Vangedal S. (1966). Fusidic acid derivatives. I. Relationship between structure and antibacterial activity. J. Med. Chem. 9, 15–22. 10.1021/jm00319a004 4959882

[B17] Kong F. Huang X. Ma Q. Xie Q. Wang P. Chen P. (2018). Helvolic acid derivatives with antibacterial activities against Streptococcus agalactiae from the marine-derived fungus Aspergillus fumigatus HNMF0047. J. Nat. Prod. 81, 1869–1876. 10.1021/acs.jnatprod.8b00382 30070829

[B18] Lannergård J. Norström T. Hughes D. (2009). Genetic determinants of resistance to fusidic acid among clinical bacteremia isolates of Staphylococcus aureus. Antimicrob. Agents Chemother. 53, 2059–2065. 10.1128/AAC.00871-08 19289529 PMC2681530

[B19] Lu J. Ni J. Wang J. Liu Z. Shang K. Bi Y. (2019). Integration of multiscale molecular modeling approaches with the design and discovery of fusidic acid derivatives. Future Med. Chem. 11, 1427–1442. 10.4155/fmc-2018-0567 31304828

[B20] Luangtongkum T. Morishita T. Y. El-Tayeb A. B. Ison A. J. Zhang Q. (2007). Comparison of antimicrobial susceptibility testing of Campylobacter spp. by the agar dilution and the agar disk diffusion methods. J. Clin. Microbiol. 45, 590–594. 10.1128/JCM.00986-06 17122005 PMC1829028

[B21] Lv J.-M. Hu D. Gao H. Kushiro T. Awakawa T. Chen G.-D. (2017). Biosynthesis of helvolic acid and identification of an unusual C-4-demethylation process distinct from sterol biosynthesis. Nat. Commun. 8, 1644. 10.1038/s41467-017-01813-9 29158519 PMC5696383

[B22] Mouchlis V. D. Melagraki G. Zacharia L. C. Afantitis A. (2020). Computer-aided drug design of β-secretase, γ-secretase and anti-tau inhibitors for the discovery of novel alzheimer's therapeutics. Int. J. Mol. Sci. 21, 703. 10.3390/ijms21030703 31973122 PMC7038192

[B23] Niu Y. Ma C. Jin H. Xu F. Gao H. Liu P. (2012). The discovery of novel beta-secretase inhibitors: Pharmacophore modeling, virtual screening, and docking studies. Chem. Biol. Drug Des. 79, 972–980. 10.1111/j.1747-0285.2012.01367.x 22381116

[B24] Petrosillo N. Granata G. Cataldo M. A. (2018). Novel antimicrobials for the treatment of clostridium difficile infection. Front. Med. 5, 96. 10.3389/fmed.2018.00096 PMC5911476 29713630

[B25] Ragab A. E. Ibrahim A.-R. S. Leon F. (2020). 3-O-formyl -27-hydroxyfusidic acid: A new metabolite of fusidic acid by cunninghamella echinulata. Rec. Nat. Prod. 14, 292–296. 10.25135/rnp.168.19.12.1505

[B26] Riber D. Venkataramana M. Sanyal S. Duvold T. (2006). Synthesis and biological evaluation of photoaffinity labeled fusidic acid analogues. J. Med. Chem. 49, 1503–1505. 10.1021/jm050583t 16509567

[B27] Sader H. S. Fritsche T. R. Jones R. N. (2006). Daptomycin bactericidal activity and correlation between disk and broth microdilution method results in testing of Staphylococcus aureus strains with decreased susceptibility to vancomycin. Antimicrob. Agents Chemother. 50, 2330–2336. 10.1128/AAC.01491-05 16801409 PMC1489799

[B28] Salimova E. V. Mamaev A. G. Tret’yakova E. V. Kukovinets O. S. Mavzyutov A. R. Shvets K. Y. (2018). Synthesis and biological activity of cyanoethyl derivatives of fusidic acid. Russ. J. Org. Chem. 54, 1411–1418. 10.1134/S1070428018090245

[B29] Sangeetha K. Sasikala R. P. Meena K. S. (2017). Pharmacophore modeling, virtual screening and molecular docking of ATPase inhibitors of HSP70. Comput. Biol. Chem. 70, 164–174. 10.1016/j.compbiolchem.2017.05.011 28910705

[B30] Schwartz C. Raible J. Mott K. Dussault P. H. (2006). Fragmentation of carbonyl oxides by N-oxides: An improved approach to alkene ozonolysis. Org. Lett. 8, 3199–3201. 10.1021/ol061001k 16836365

[B31] Sharma T. Harioudh M. K. Kuldeep J. Kumar S. Banerjee D. Ghosh J. K. (2020). Identification of potential inhibitors of cathepsin-B using shape & pharmacophore-based virtual screening, molecular docking and explicit water thermodynamics. Mol. Inf. 39, 1900023. 10.1002/minf.201900023 31648416

[B32] Singh K. Kaur G. Shanika P. S. Dziwornu G. A. Okombo J. Chibale K. (2020). Structure-activity relationship analyses of fusidic acid derivatives highlight crucial role of the C-21 carboxylic acid moiety to its anti-mycobacterial activity. Bioorg. Med. Chem. 28, 115530. 10.1016/j.bmc.2020.115530 32362386

[B33] Talambedu U. Dhivya S. Arvind Kumar G. Chinaga Suresh K. Sushil Kumar M. (2017). Recent updates on computer-aided drug discovery: Time for a paradigm shift. Curr. Top. Med. Chem. 17, 3296–3307. 10.2174/1568026618666180101163651 29295698

[B34] Tanaka N. Kinoshita T. Masukawa H. (1968). Mechanism of protein synthesis inhibition by fusidic acid and related antibiotics. Biochem. Biophysical Res. Commun. 30, 278–283. 10.1016/0006-291X(68)90447-6 4296678

[B35] Turnidge J. (1999). Fusidic acid pharmacology, pharmacokinetics and pharmacodynamics. Int. J. Antimicrob. Agents 12, S23–S34. 10.1016/S0924-8579(98)00071-5 10528784

[B36] Von Daehne W. Godtfredsen W. Rasmussen P. (1979). Structure-activity relationships in fusidic acid-type antibiotics. Adv. Appl. Microbiol. 25, 95–146. 10.1016/s0065-2164(08)70148-5 397741

[B37] Wang M. Zhu H. Wang P. Zeng D. Wu Y. Liu L. (2019). Synthesis of thiazolium-labeled 1, 3, 4-oxadiazole thioethers as prospective antimicrobials: *In vitro* and *in vivo* bioactivity and mechanism of action. J. Agric. Food Chem. 67, 12696–12708. 10.1021/acs.jafc.9b03952 31657554

[B38] Zhao M. Godecke T. Gunn J. Duan J. A. Che C. T. (2013). Protostane and fusidane triterpenes: A mini-review. Molecules 18, 4054–4080. 10.3390/molecules18044054 23563857 PMC3901436

[B39] Zhao S. Wu P. Zhang K. Hong W. Jiang Z. Cui X. (2016). Chemically modified fusidic acid, preparation method and application. Guangdong: China National Intellectual Property Administration, C. N. Patent No 105,924,488.

[B40] Zhu C. Li X. W. Zhao B. Y. Peng W. Q. Li W. Fu W. (2020). Discovery of aryl-piperidine derivatives as potential antipsychotic agents using molecular hybridization strategy. Eur. J. Med. Chem. 193, 112214. 10.1016/j.ejmech.2020.112214 32182489

