# Supplementary material for: Ligand and structure-based approaches for the exploration of structure–activity relationships of fusidic acid derivatives as antibacterial agents
Source: Front Chem. 2023 Jan 6;10:1094841. doi: 10.3389/fchem.2022.1094841 (PMC9852990; doi:10.3389/fchem.2022.1094841)
Supplement: Supplementary file 1 [file DataSheet1.PDF]

Supplementary Material

Original ^{1}H, ^{13}C NMR and ESI-HRMS spectra of target compounds.

^{1}H NMR (400 MHz, $CDCl_3$) δ 7.27 (s, 1H), 5.86 (d, J = 8.4 Hz, 1H), 5.74 (dd, 2H), 5.08 (t, J = 7.0 Hz, 1H), 4.34 (s, 1H), 3.74 (d, J = 8.3 Hz, 1H), 3.05 (d, J = 11.1 Hz, 1H), 2.54 – 2.36 (m, 2H), 2.31 (d, J = 13.1 Hz, 1H), 2.25 – 2.06 (m, 4H), 1.98 (s, 3H), 1.93 – 1.79 (m, 2H), 1.79 – 1.70 (m, 2H), 1.67 (s, 3H), 1.61 – 1.47 (m, 8H), 1.37 (s, 3H), 1.29 (t, J = 16.0, 7.6 Hz, 2H), 1.21 (s, 9H), 1.18 – 1.02 (m, 2H), 0.97 (s, 3H), 0.90 (d, J = 16.7, 7.5 Hz, 6H).

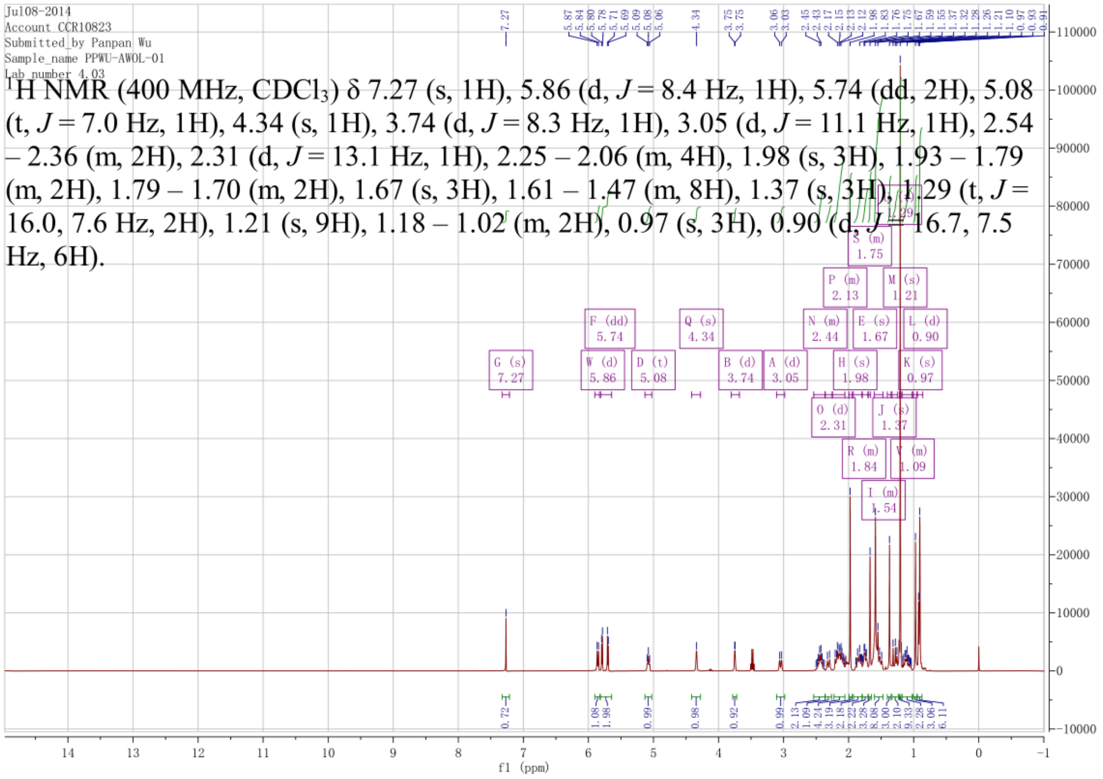

^{1}H NMR of compound **FA-1**.

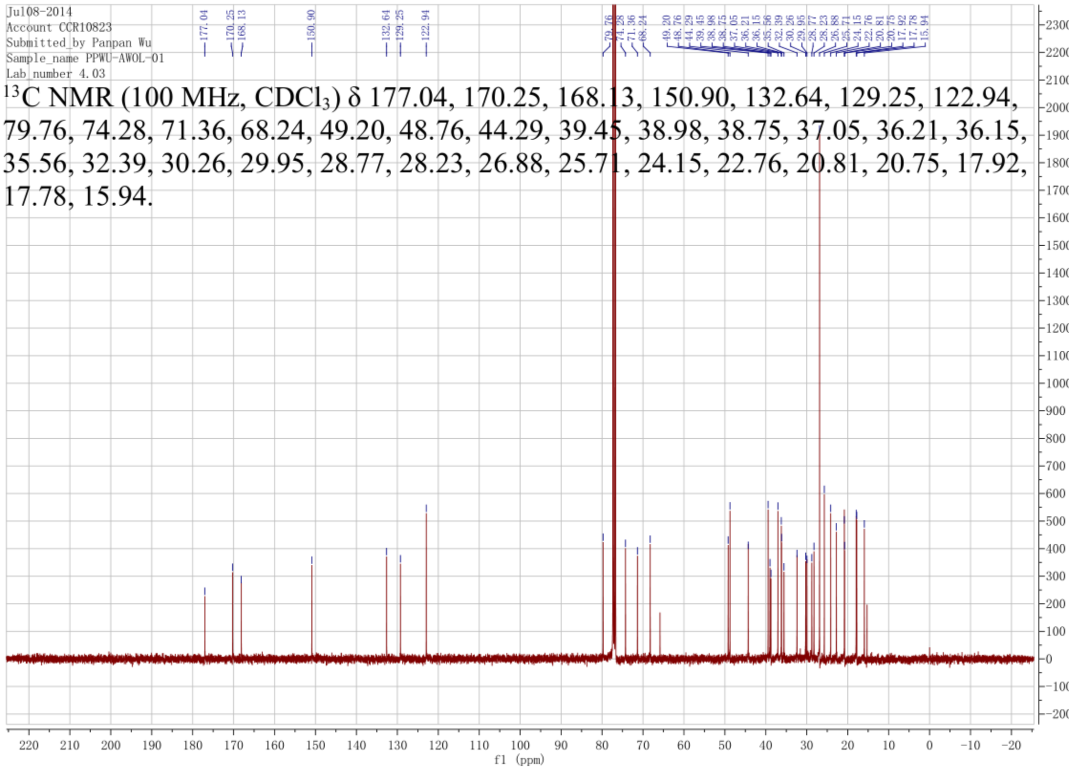

^{13}C NMR of compound **FA-1**.

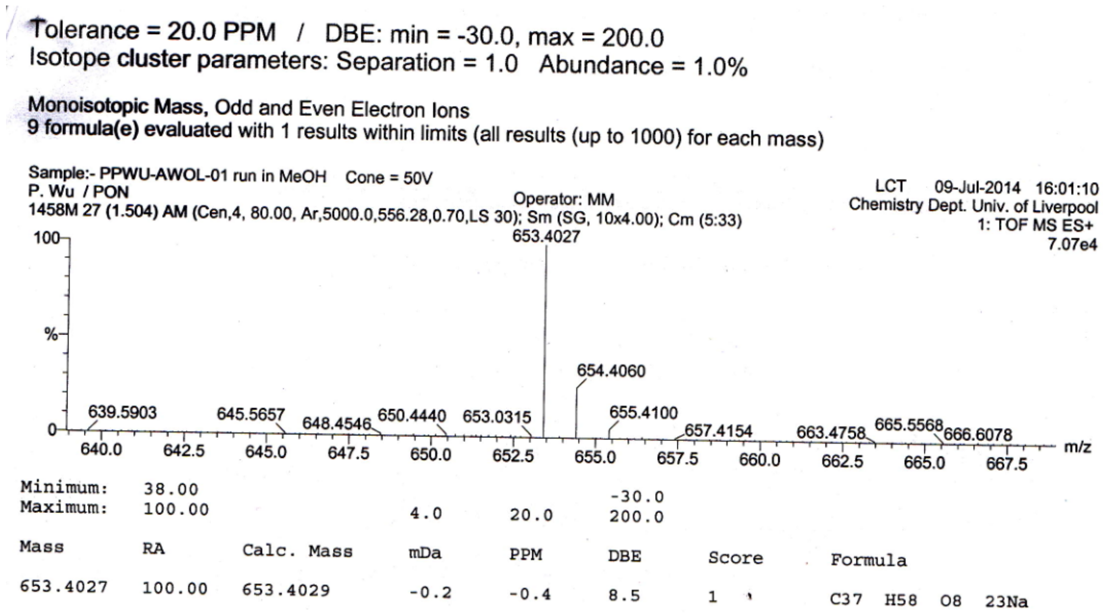

HRMS of compound **FA-1**.

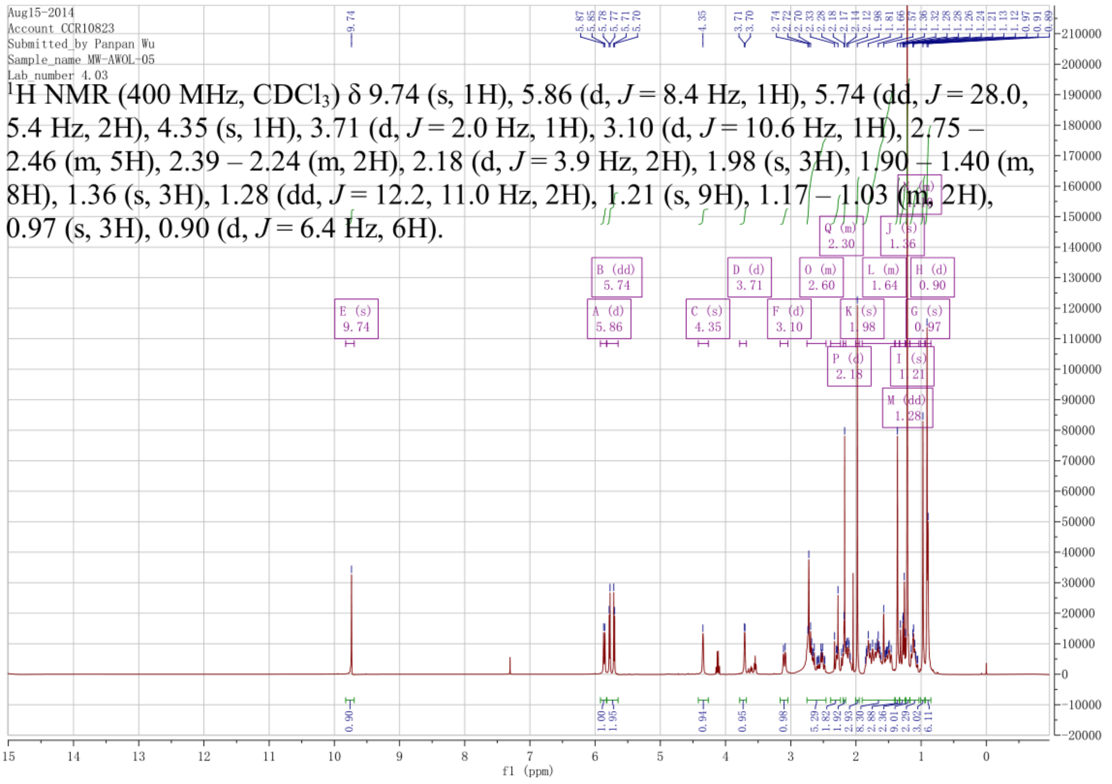

^{1}H NMR of compound **FA-2**.

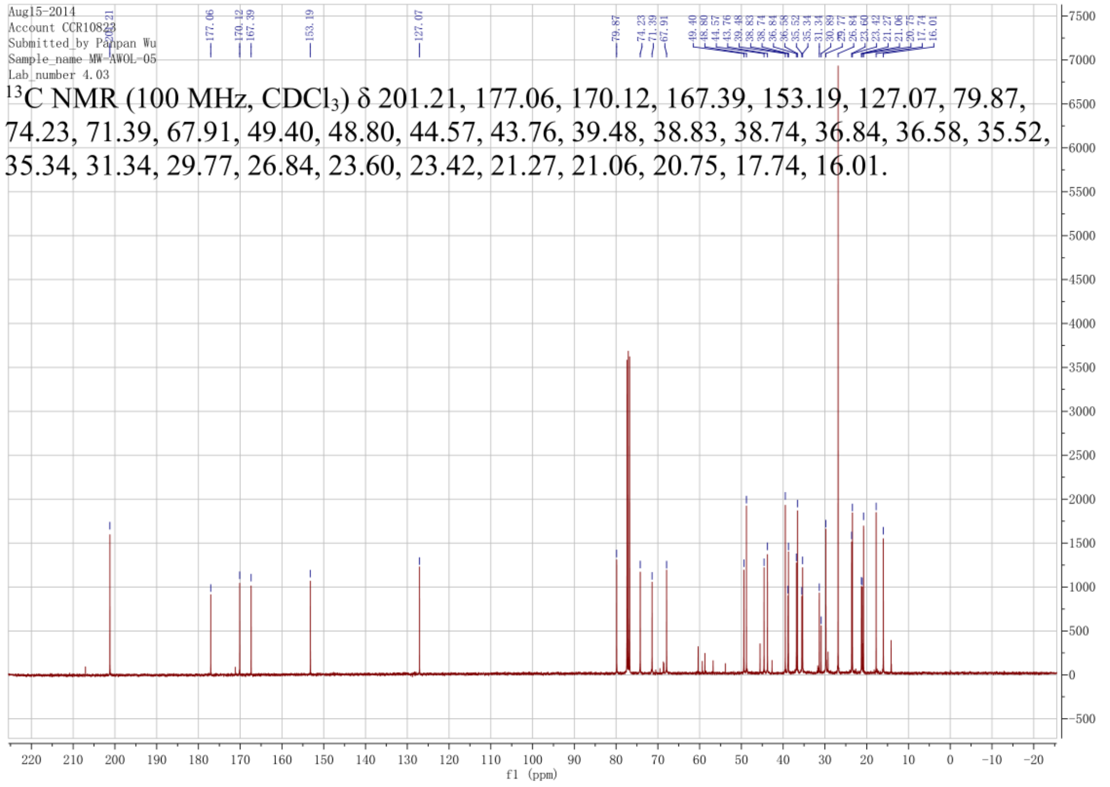

^{13}C NMR of compound **FA-2**.

Tolerance = 20.0 PPM / DBE: min = -30.0, max = 200.0
Isotope cluster parameters: Separation = 1.0 Abundance = 1.0%

Monoisotopic Mass, Odd and Even Electron Ions
12 formula(e) evaluated with 1 results within limits (all results (up to 1000) for each mass)

Sample:- PPWU-FA-07 run in MeOH Cone = 60V
P. Wu / PON
587N 23 (1.284) AM (Cen,4, 80.00, Ar,5000.0,556.28,0.70,LS 1); Sm (SG, 10x2.00); Cm (5:33)
Operator: MM
LCT 29-Jun-2015 16:08:56
Chemistry Dept. Univ. of Liverpool
1: TOF MS ES+
2.27e4

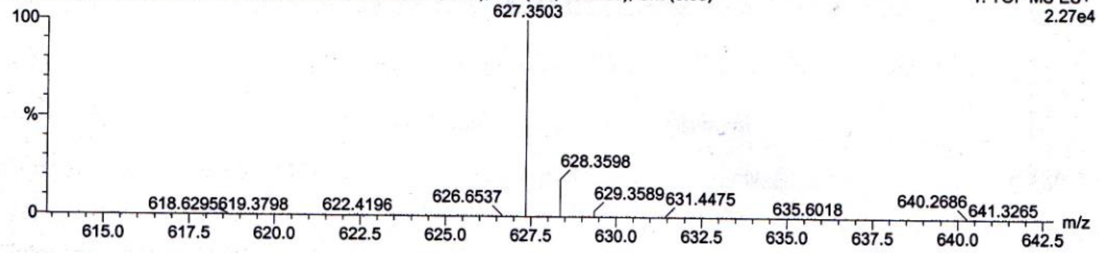

Minimum:	60.00				-30.0		
Maximum:	100.00		4.0	20.0	200.0		
Mass	RA	Calc. Mass	mDa	PPM	DBE	Score	Formula
627.3503	100.00	627.3509	-0.6	-1.0	8.5	1	C34 H52 O9 23Na

HRMS of compound **FA-2**.

Tolerance = 20.0 PPM / DBE: min = -30.0, max = 200.0
Isotope cluster parameters: Separation = 1.0 Abundance = 1.0%

Monoisotopic Mass, Odd and Even Electron Ions
10 formula(e) evaluated with 1 results within limits (all results (up to 1000) for each mass)

Sample:- PPWU-AWOL-14 run in MeOH Cone = 50V
P. Wu / PON
1569M 15 (0.808) AM (Cen,4, 80.00, Ar,5000.0,556.28,0.70,LS 1); Sm (SG, 10x4.00); Cm (3:37)
Operator: MM
LCT 19-Aug-2014 13:38:20
Chemistry Dept. Univ. of Liverpool
1: TOF MS ES+
3.69e4

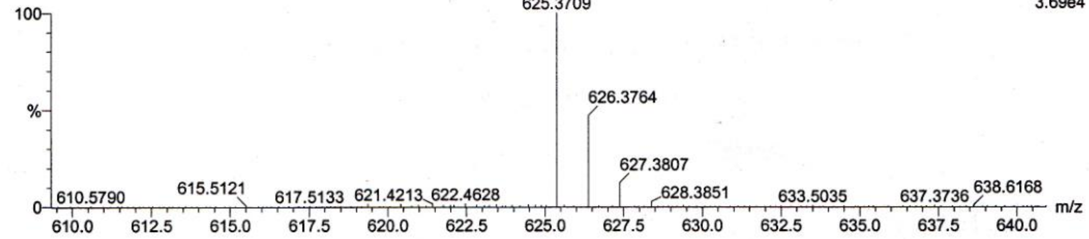

Minimum:	48.00				-30.0		
Maximum:	100.00		4.0	20.0	200.0		
Mass	RA	Calc. Mass	mDa	PPM	DBE	Score	Formula
625.3709	100.00	625.3716	-0.7	-1.2	8.5	1	C35 H54 O8 23Na

HRMS of compound **FA-3**.

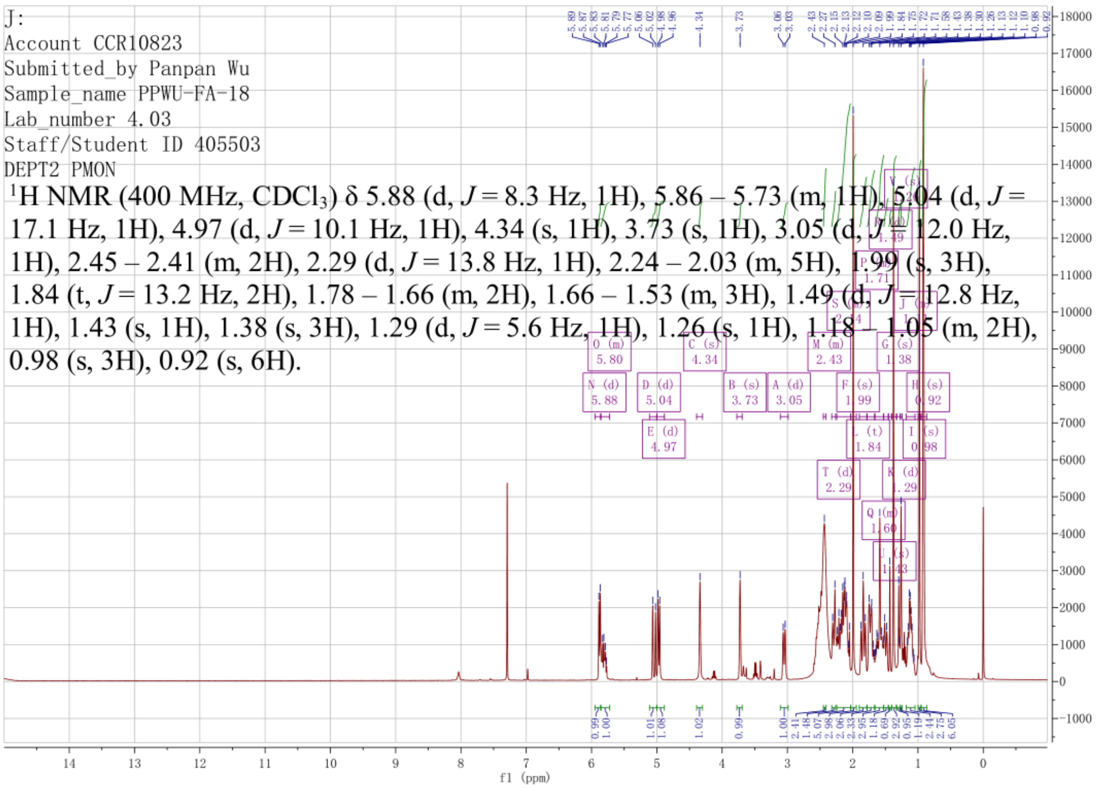

^{1}H NMR of compound **FA-6**.

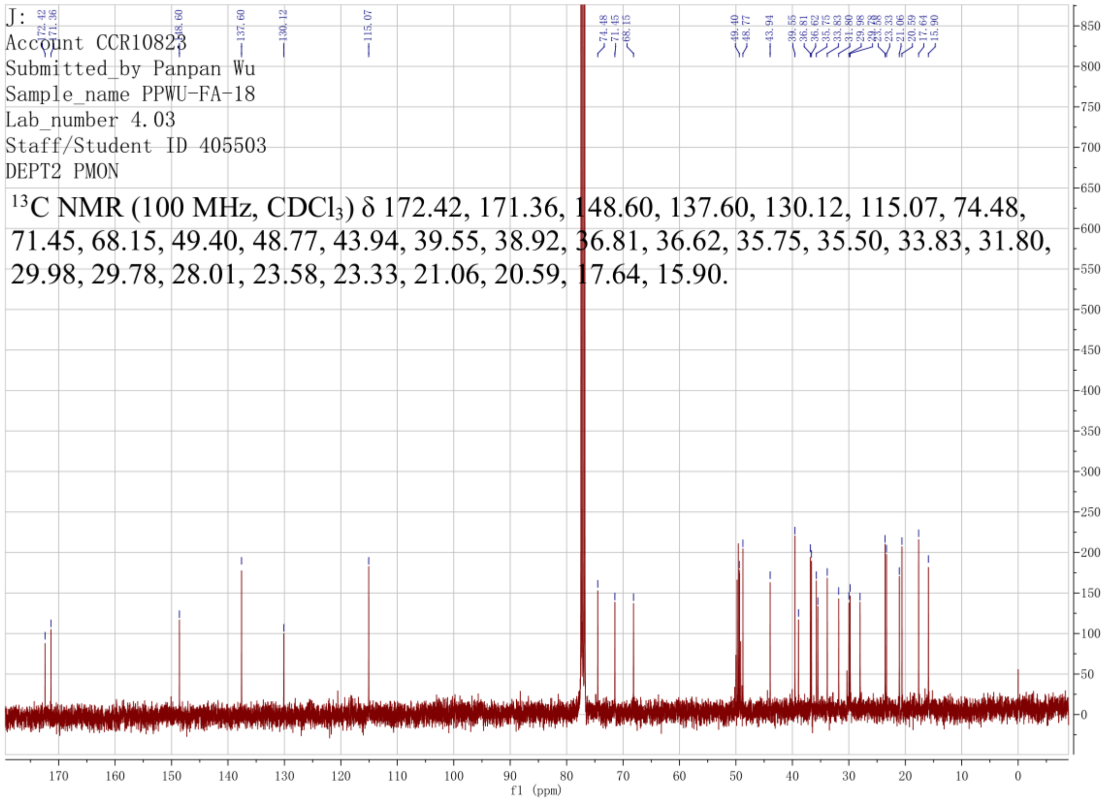

^{13}C NMR of compound **FA-6**.

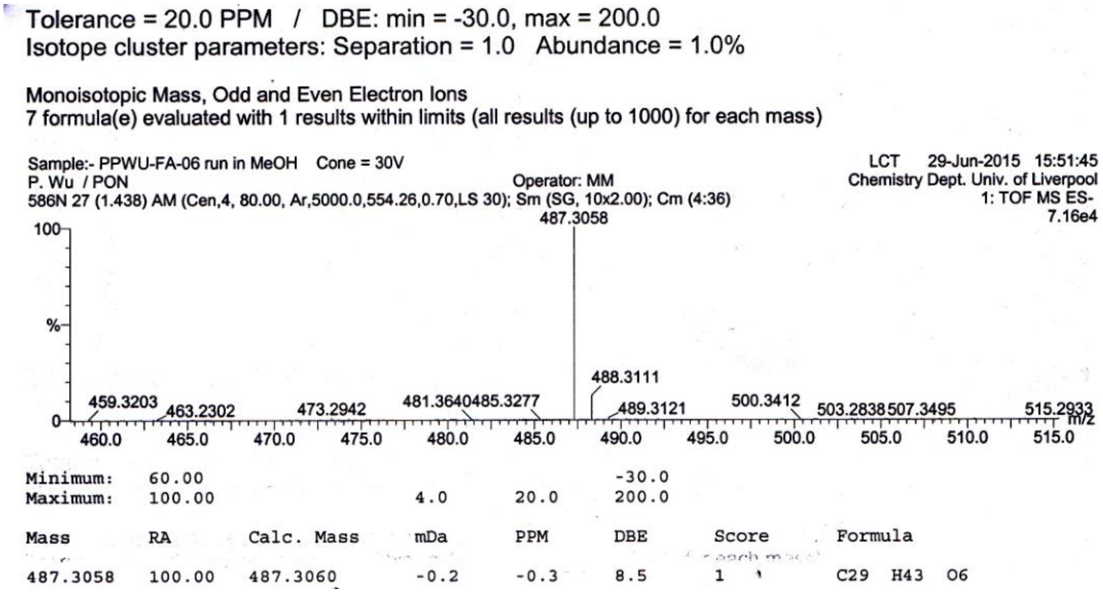

HRMS of compound **FA-6**.

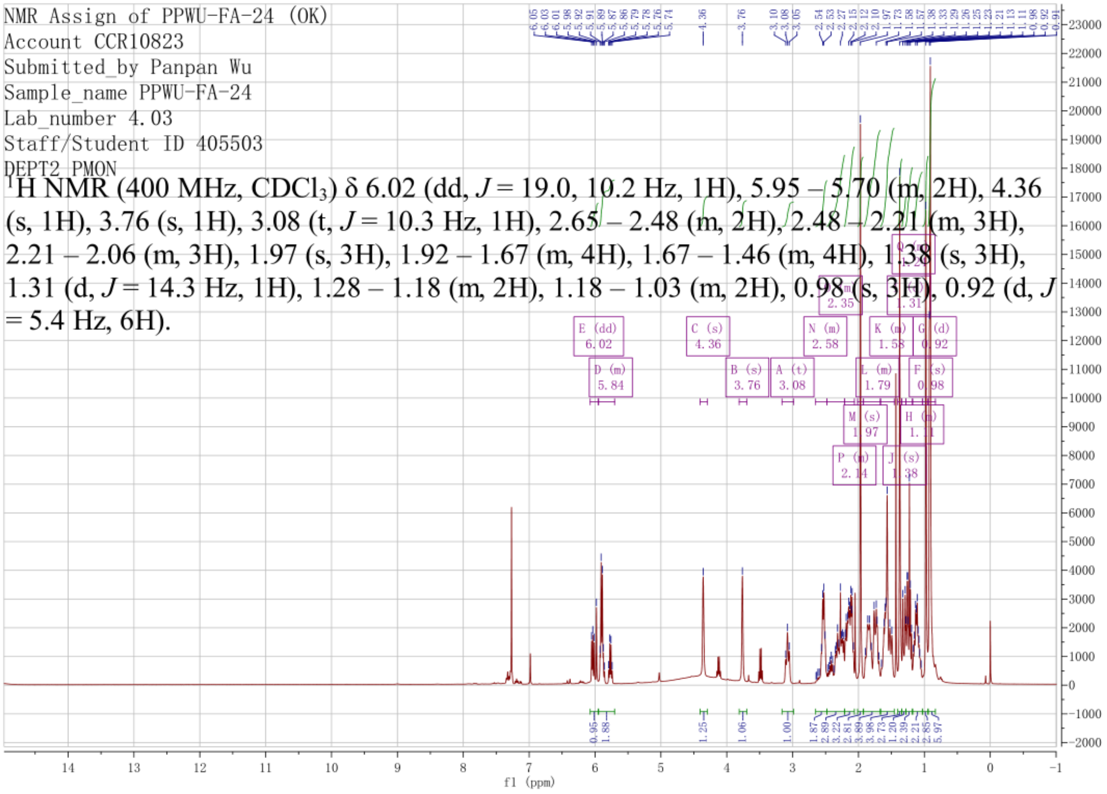

^{1}H NMR (400 MHz, $CDCl_3$) δ 6.02 (dd, *J* = 19.0, 10.2 Hz, 1H), 5.95 – 5.70 (m, 2H), 4.36 (s, 1H), 3.76 (s, 1H), 3.08 (t, *J* = 10.3 Hz, 1H), 2.65 – 2.48 (m, 2H), 2.48 – 2.21 (m, 3H), 2.21 – 2.06 (m, 3H), 1.97 (s, 3H), 1.92 – 1.67 (m, 4H), 1.67 – 1.46 (m, 4H), 1.38 (s, 3H), 1.31 (d, *J* = 14.3 Hz, 1H), 1.28 – 1.18 (m, 2H), 1.18 – 1.03 (m, 2H), 0.98 (s, 3H), 0.92 (d, *J* = 5.4 Hz, 6H).

^{1}H NMR of compound **FA-7**.

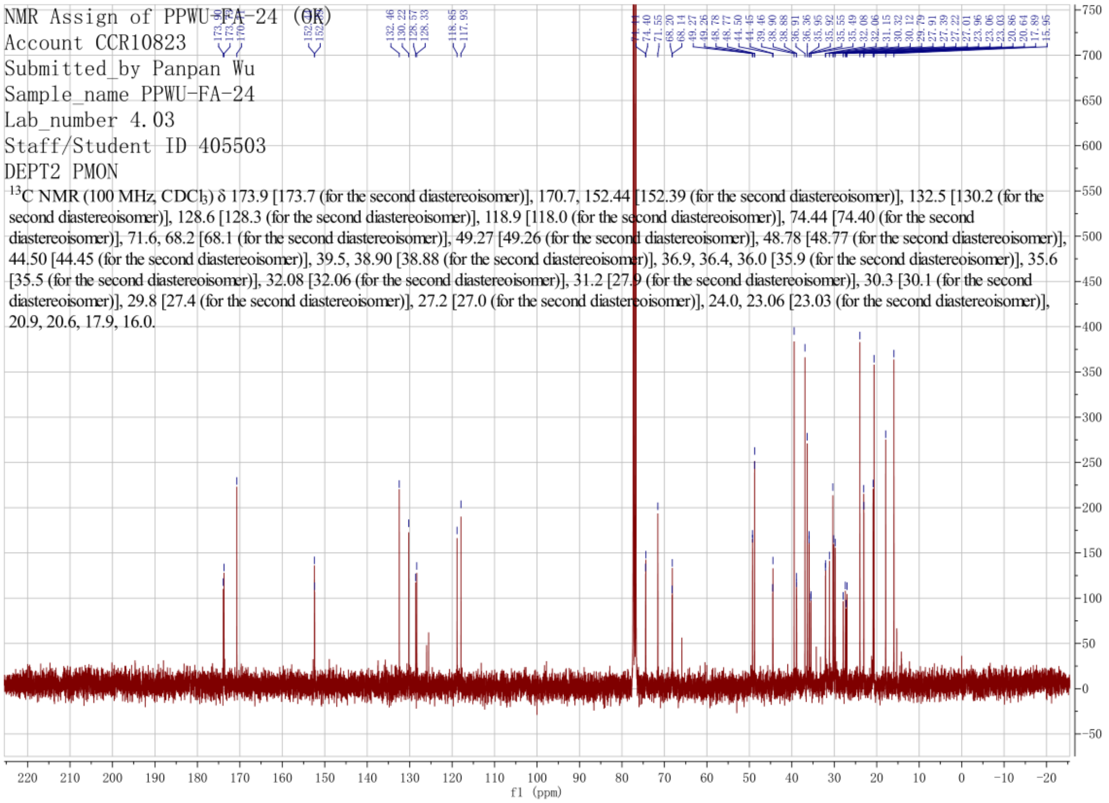

^{13}C NMR of compound **FA-7**.

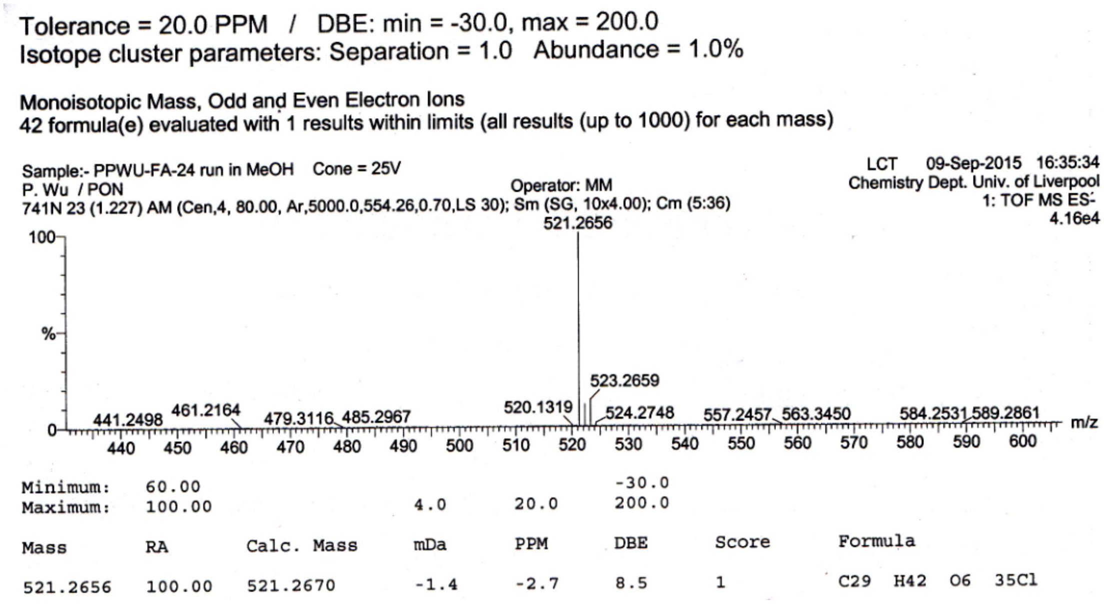

HRMS of compound **FA-7**.

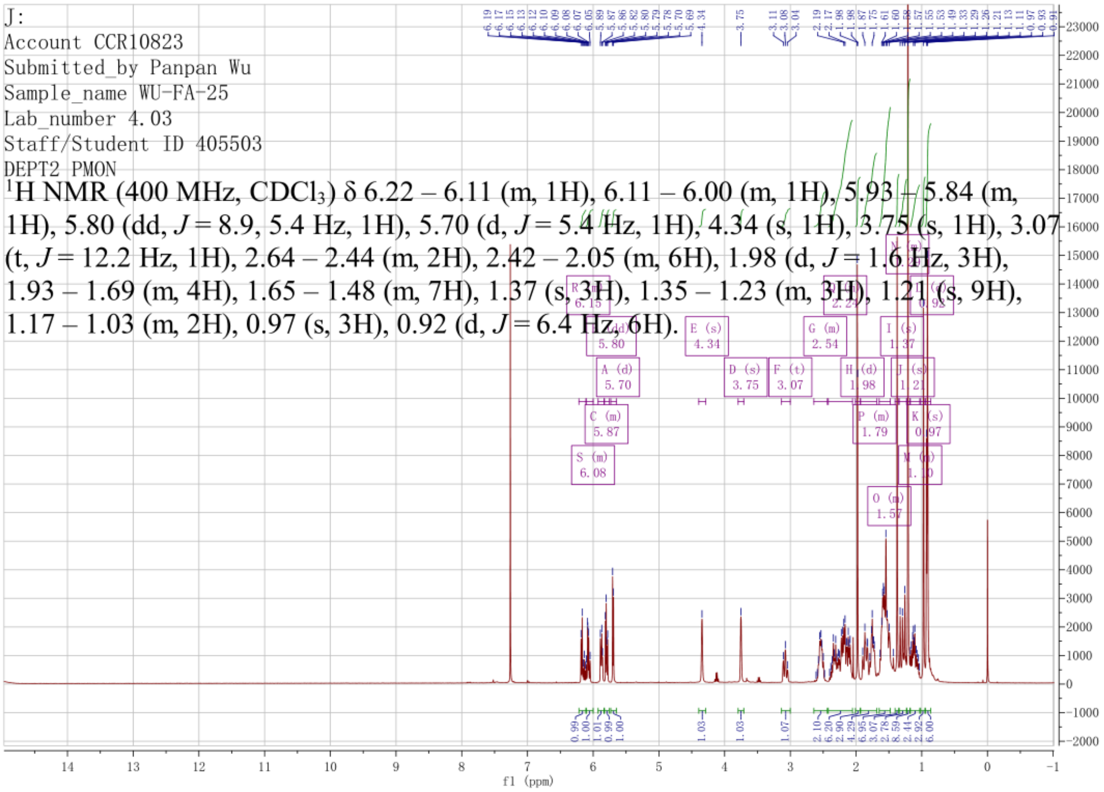

^{1}H NMR of compound **FA-8**.

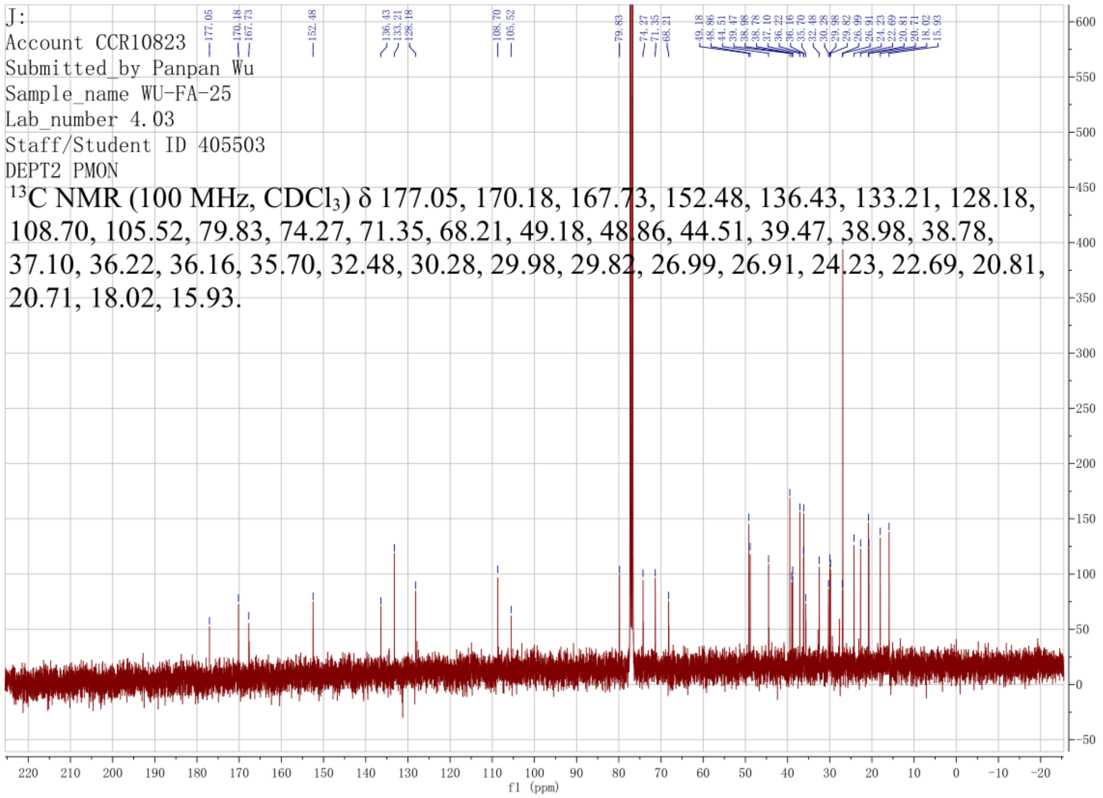

^{13}C NMR of compound **FA-8**.

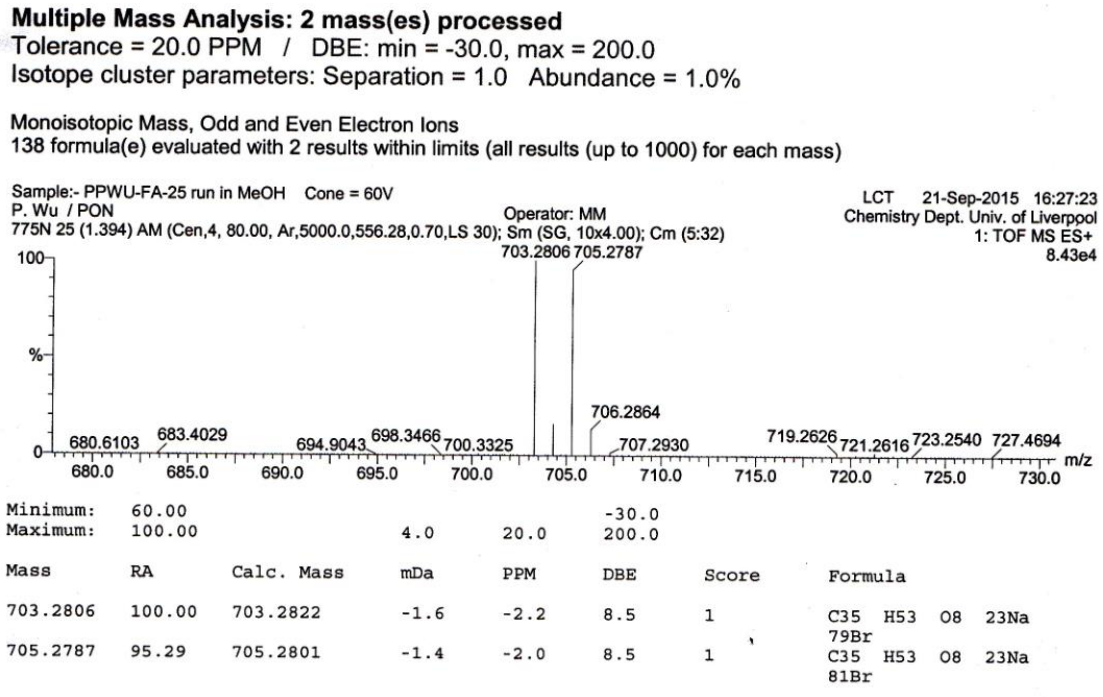

Mass	RA	Calc. Mass	mDa	PPM	DBE	Score	Formula
703.2806	100.00	703.2822	-1.6	-2.2	8.5	1	C35 H53 O8 23Na 79Br
705.2787	95.29	705.2801	-1.4	-2.0	8.5	1	C35 H53 O8 23Na 81Br

HRMS of compound **FA-8**.

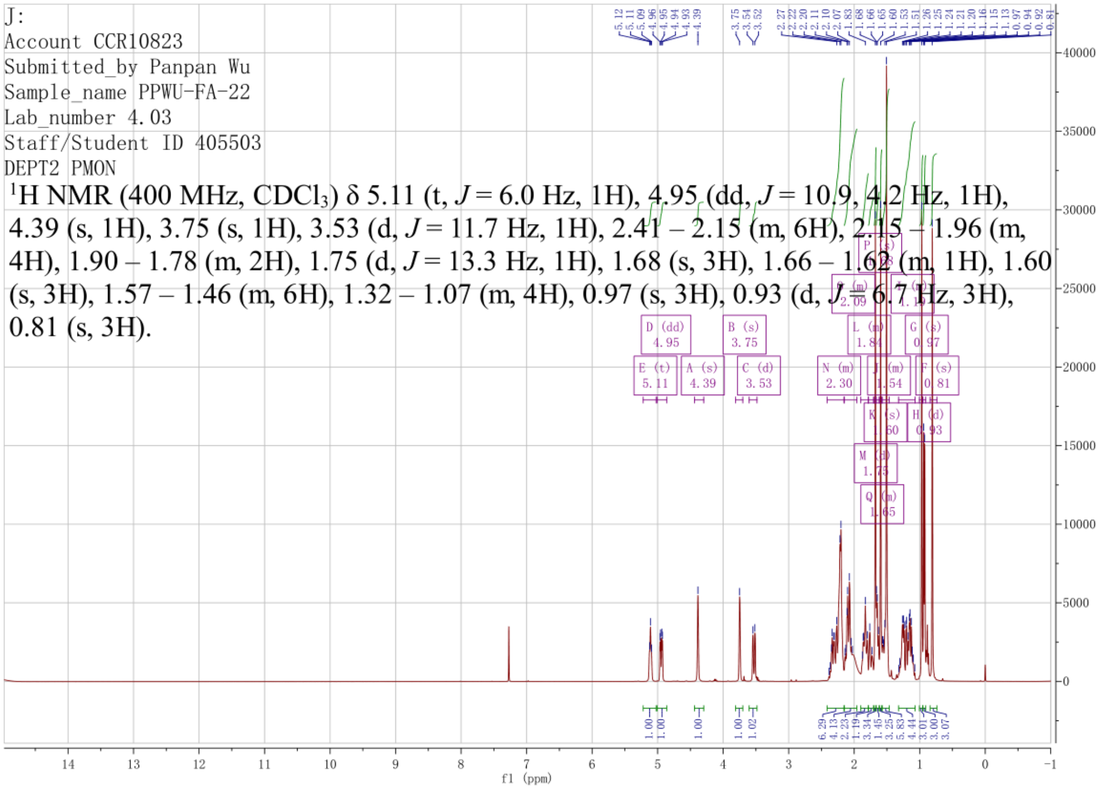

^{1}H NMR of compound **FA-9**.

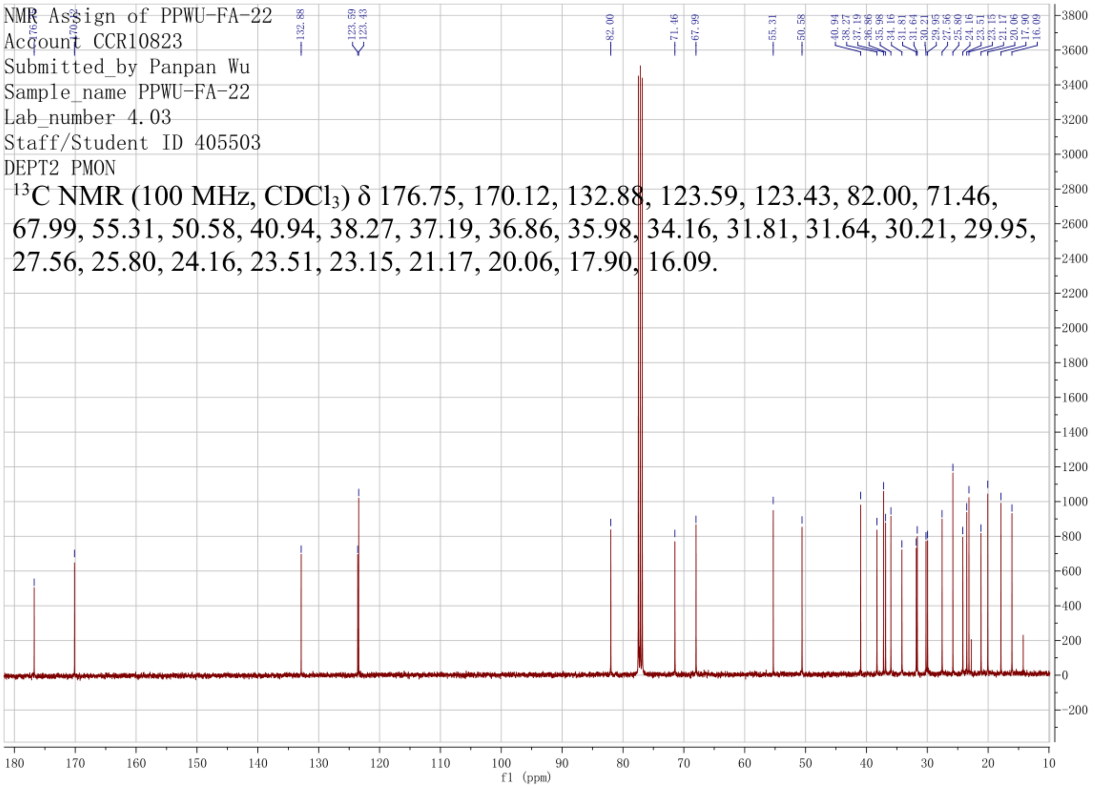

^{13}C NMR of compound **FA-9**.

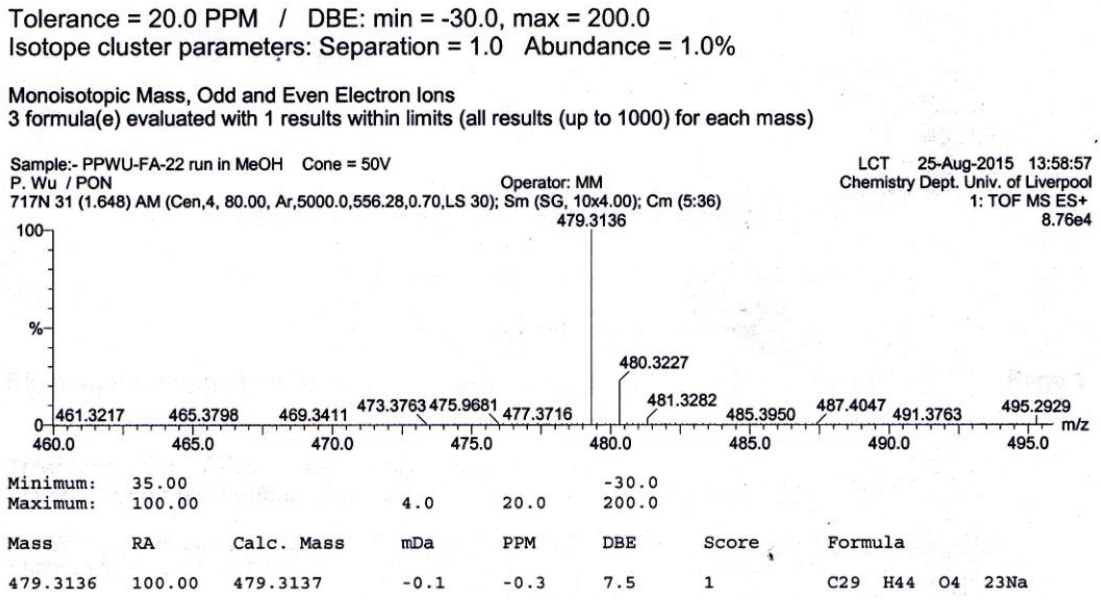

HRMS of compound **FA-9**.

Tolerance = 20.0 PPM / DBE: min = -30.0, max = 200.0
Isotope cluster parameters: Separation = 1.0 Abundance = 1.0%

Monoisotopic Mass, Odd and Even Electron Ions
29 formula(e) evaluated with 1 results within limits (all results (up to 1000) for each mass)

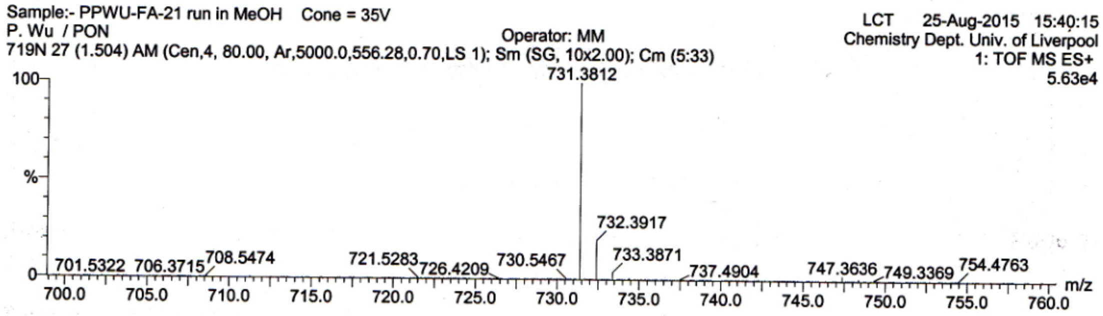

Minimum:	35.00				-30.0		
Maximum:	100.00		4.0	20.0	200.0		
Mass	RA	Calc. Mass	mDa	PPM	DBE	Score	Formula
731.3812	100.00	731.3805	0.7	1.0	8.5	1	C38 H60 O10 23Na S

HRMS of compound **FA-10**.

Tolerance = 20.0 PPM / DBE: min = -30.0, max = 200.0
Isotope cluster parameters: Separation = 1.0 Abundance = 1.0%

Monoisotopic Mass, Odd and Even Electron Ions
36 formula(e) evaluated with 1 results within limits (all results (up to 1000) for each mass)

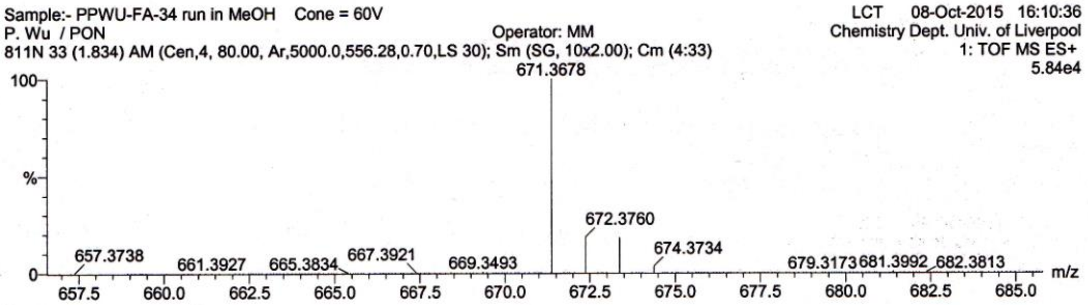

Minimum:	30.00				-30.0		
Maximum:	100.00		4.0	20.0	200.0		
Mass	RA	Calc. Mass	mDa	PPM	DBE	Score	Formula
671.3678	100.00	671.3691	-1.3	-1.9	8.5	1	C37 H57 O7 23Na 35Cl

HRMS of compound **FA-11**.

Multiple Mass Analysis: 2 mass(es) processed
Tolerance = 20.0 PPM / DBE: min = -30.0, max = 200.0
Isotope cluster parameters: Separation = 1.0 Abundance = 1.0%

Monoisotopic Mass, Odd and Even Electron Ions
124 formula(e) evaluated with 2 results within limits (all results (up to 1000) for each mass)

Sample:- PPWU-FA-27 run in MeOH Cone = 50V | LCT 12-Oct-2015 16:26:07
P. Wu / PON | Operator: MM | Chemistry Dept. Univ. of Liverpool
822N 33 (1.835) AM (Cen,4, 80.00, Ar,5000.0,556.28,0.70,LS 30); Sm (SG, 10x4.00); Cm (3:34) | 1: TOF MS ES+ | 4.79e4

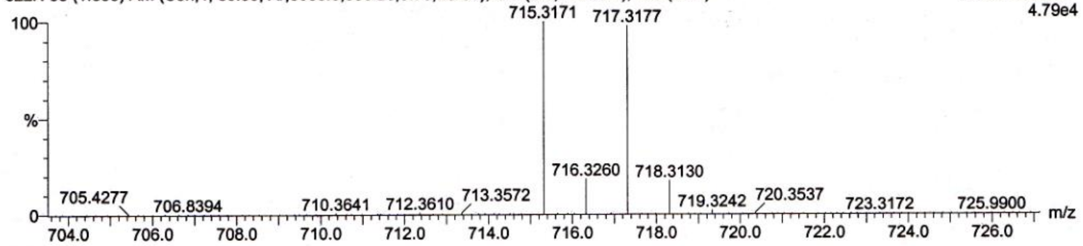

Mass	RA	Calc. Mass	mDa	PPM	DBE	Score	Formula
Minimum:	30.00				-30.0		
Maximum:	100.00		4.0	20.0	200.0		
715.3171	100.00	715.3185	-1.4	-2.0	8.5	1	C37 H57 O7 23Na 79Br
717.3177	97.75	717.3165	1.2	1.7	8.5	1	C37 H57 O7 23Na 81Br

HRMS of compound **FA-12**.

Tolerance = 20.0 PPM / DBE: min = -30.0, max = 200.0
Isotope cluster parameters: Separation = 1.0 Abundance = 1.0%

Monoisotopic Mass, Odd and Even Electron Ions
23 formula(e) evaluated with 1 results within limits (all results (up to 1000) for each mass)

Sample:- PPWU-FA-28 run in MeOH Cone = 50V | LCT 16-Oct-2015 12:21:10
P. Wu / PON | Operator: MM | Chemistry Dept. Univ. of Liverpool
831N 29 (1.614) AM (Cen,4, 80.00, Ar,5000.0,556.28,0.70,LS 30); Sm (SG, 10x2.00); Cm (2:33) | 1: TOF MS ES+ | 6.69e4

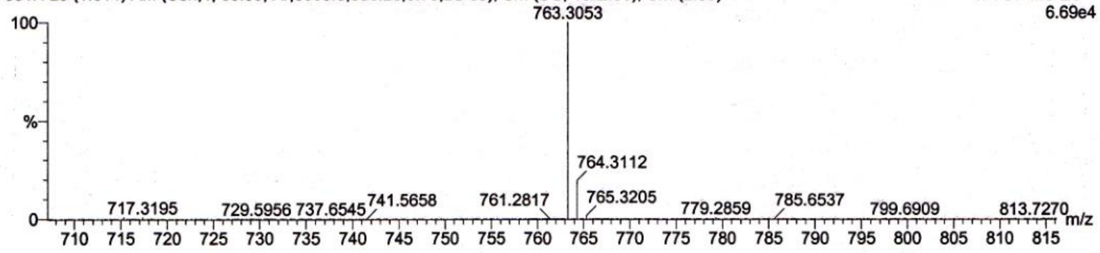

Mass	RA	Calc. Mass	mDa	PPM	DBE	Score	Formula
Minimum:	30.00				-30.0		
Maximum:	100.00		4.0	20.0	200.0		
763.3053	100.00	763.3047	0.6	0.8	8.5	1	C37 H57 O7 23Na 127I

HRMS of compound **FA-13**.

Tolerance = 20.0 PPM / DBE: min = -30.0, max = 200.0
Isotope cluster parameters: Separation = 1.0 Abundance = 1.0%

Monoisotopic Mass, Odd and Even Electron Ions
36 formula(e) evaluated with 1 results within limits (all results (up to 1000) for each mass)

Sample:- PPWU-FA-33 run in MeOH Cone = 50V | LCT 12-Oct-2015 16:08:53
P. Wu / PON | Operator: MM | Chemistry Dept. Univ. of Liverpool
821N 19 (1.064) AM (Cen,4, 80.00, Ar,5000.0,556.28,0.70,LS 30); Sm (SG, 10x2.00); Cm (5:34) | 1: TOF MS ES+ | 8.27e4

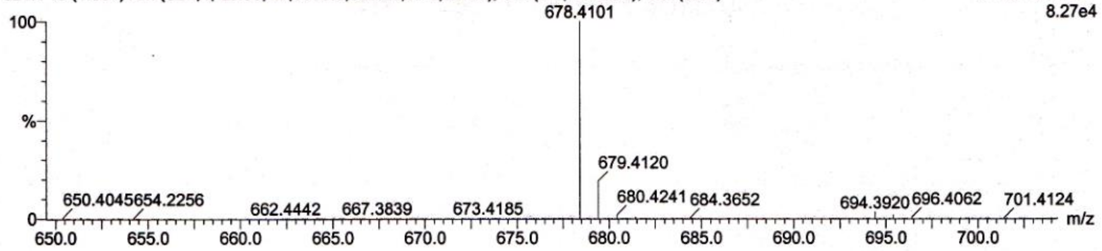

Minimum:	30.00				-30.0		
Maximum:	100.00		4.0	20.0	200.0		
Mass	RA	Calc. Mass	mDa	PPM	DBE	Score	Formula
678.4101	100.00	678.4094	0.7	1.0	10.5	1	C37 H57 N3 O7 23Na

HRMS of compound **FA-14**.

Tolerance = 20.0 PPM / DBE: min = -30.0, max = 200.0
Isotope cluster parameters: Separation = 1.0 Abundance = 1.0%

Monoisotopic Mass, Odd and Even Electron Ions
23 formula(e) evaluated with 1 results within limits (all results (up to 1000) for each mass)

Sample:- PPWU-FA-29 run in MeOH Cone = 50V | LCT 27-Oct-2015 16:52:14
P. Wu / PON | Operator: MM | Chemistry Dept. Univ. of Liverpool
859N 33 (1.753) AM (Cen,4, 80.00, Ar,5000.0,556.28,0.70,LS 30); Sm (SG, 10x2.00); Cm (3:36) | 1: TOF MS ES+ | 1.41e4

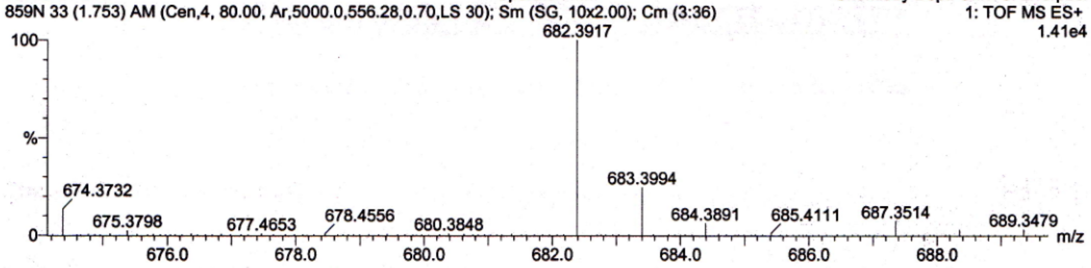

Minimum:	30.00				-30.0		
Maximum:	100.00		4.0	20.0	200.0		
Mass	RA	Calc. Mass	mDa	PPM	DBE	Score	Formula
682.3917	100.00	682.3931	-1.4	-2.1	9.5	1	C37 H57 N O9 23Na

HRMS of compound **FA-15**.

Tolerance = 20.0 PPM / DBE: min = -30.0, max = 200.0
Isotope cluster parameters: Separation = 1.0 Abundance = 1.0%

Monoisotopic Mass, Odd and Even Electron Ions
11 formula(e) evaluated with 1 results within limits (all results (up to 1000) for each mass)

Sample:- PPWU-FA-39 run in MeOH Cone = 40V LCT 16-Oct-2015 17:05:07
P. Wu / PON Operator: MM Chemistry Dept. Univ. of Liverpool
840N 25 (1.394) AM (Cen,4, 80.00, Ar,5000.0,556.28,0.70,LS 1); Sm (SG, 10x4.00); Cm (4:33) 1: TOF MS ES+
7.05e4

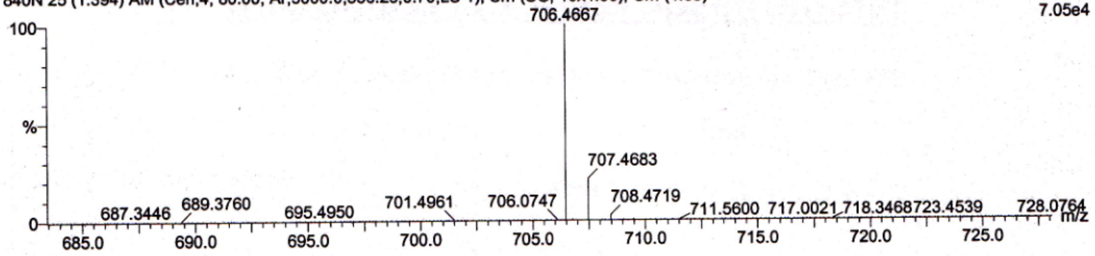

Minimum:	30.00				-30.0		
Maximum:	100.00		4.0	20.0	200.0		
Mass	RA	Calc. Mass	mDa	PPM	DBE	Score	Formula
706.4667	100.00	706.4683	-1.6	-2.2	12.5	1	C43 H64 N O7

HRMS of compound **FA-16**.

Multiple Mass Analysis: 2 mass(es) processed
Tolerance = 20.0 PPM / DBE: min = -30.0, max = 200.0
Isotope cluster parameters: Separation = 1.0 Abundance = 1.0%

Monoisotopic Mass, Odd and Even Electron Ions
26 formula(e) evaluated with 1 results within limits (all results (up to 1000) for each mass)

Sample:- PPWU-FA-35 run in MeOH Cone = 60V LCT 12-Oct-2015 17:02:37
P. Wu / PON Operator: MM Chemistry Dept. Univ. of Liverpool
823N 29 (1.543) AM (Cen,4, 80.00, Ar,5000.0,556.28,0.70,LS 1); Sm (SG, 10x4.00); Cm (5:36) 1: TOF MS ES+
3.17e4

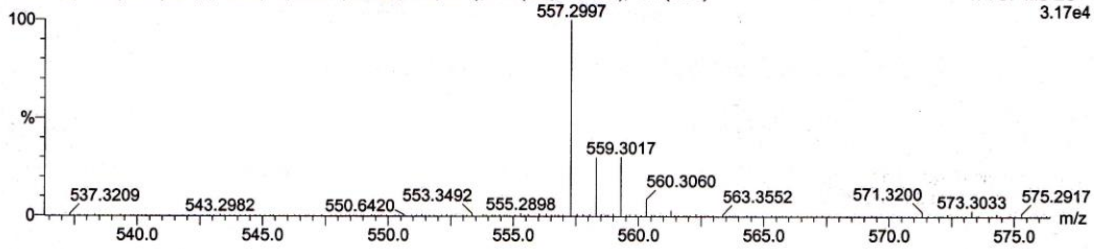

Minimum:	32.00				-30.0		
Maximum:	100.00		4.0	20.0	200.0		
Mass	RA	Calc. Mass	mDa	PPM	DBE	Score	Formula
557.2997	100.00	557.3010	-1.3	-2.3	7.5	1	C31 H47 O5 23Na 35Cl

HRMS of compound **FA-17**.

Multiple Mass Analysis: 2 mass(es) processed
Tolerance = 20.0 PPM / DBE: min = -30.0, max = 200.0
Isotope cluster parameters: Separation = 1.0 Abundance = 1.0%

Monoisotopic Mass, Odd and Even Electron Ions
60 formula(e) evaluated with 2 results within limits (all results (up to 1000) for each mass)

Sample:- PPWU-FA-36 run in MeOH Cone = 40V
P. Wu / PON
827N 10 (0.527) AM (Cen,4, 80.00, Ar,5000.0,554.26,0.70,LS 1); Sm (SG, 10x2.00); Cm (4:35)
Operator: MM
LCT 13-Oct-2015 16:09:01
Chemistry Dept. Univ. of Liverpool
1: TOF MS ES-
1.73e3

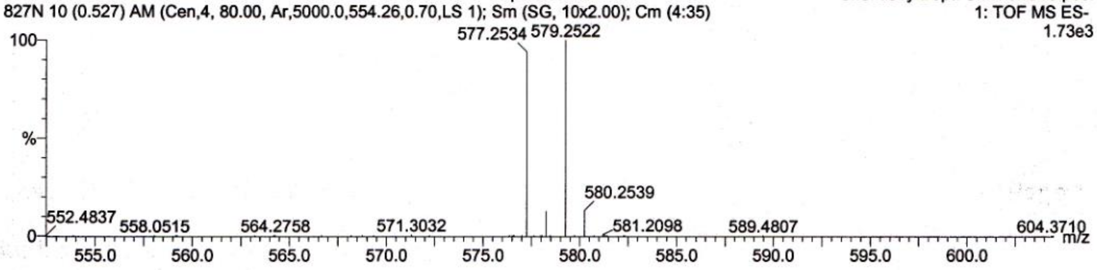

Minimum:	32.00				-30.0		
Maximum:	100.00		4.0	20.0	200.0		

Mass	RA	Calc. Mass	mDa	PPM	DBE	Score	Formula
577.2534	93.83	577.2529	0.5	0.9	8.5	1	C31 H46 O5 79Br
579.2522	100.00	579.2508	1.4	2.4	8.5	1	C31 H46 O5 81Br

HRMS of compound **FA-18**.

Tolerance = 20.0 PPM / DBE: min = -30.0, max = 200.0
Isotope cluster parameters: Separation = 1.0 Abundance = 1.0%

Monoisotopic Mass, Odd and Even Electron Ions
17 formula(e) evaluated with 1 results within limits (all results (up to 1000) for each mass)

Sample:- PPWU-FA-41 run in MeOH Cone = 30V
P. Wu / PON
846N 22 (1.158) AM (Cen,4, 80.00, Ar,5000.0,554.26,0.70,LS 30); Sm (SG, 10x2.00); Cm (4:35)
Operator: MM
LCT 21-Oct-2015 15:16:04
Chemistry Dept. Univ. of Liverpool
1: TOF MS ES-
2.13e3

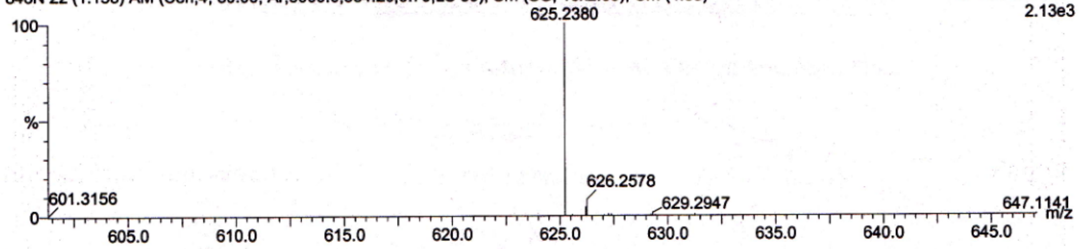

Minimum:	30.00				-30.0		
Maximum:	100.00		4.0	20.0	200.0		

Mass	RA	Calc. Mass	mDa	PPM	DBE	Score	Formula
625.2380	100.00	625.2390	-1.0	-1.6	8.5	1	C31 H46 O5 127I

HRMS of compound FA-19.

Tolerance = 20.0 PPM / DBE: min = -30.0, max = 200.0
Isotope cluster parameters: Separation = 1.0 Abundance = 1.0%

Monoisotopic Mass, Odd and Even Electron Ions
19 formula(e) evaluated with 1 results within limits (all results (up to 1000) for each mass)

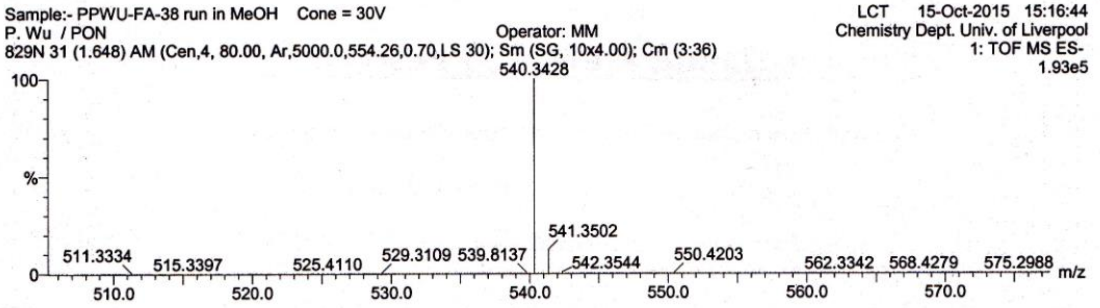

Minimum:	32.00				-30.0		
Maximum:	100.00		4.0	20.0	200.0		
Mass	RA	Calc. Mass	mDa	PPM	DBE	Score	Formula
540.3428	100.00	540.3437	-0.9	-1.8	10.5	1	C31 H46 N3 O5

HRMS of compound **FA-20**.

Tolerance = 20.0 PPM / DBE: min = -30.0, max = 200.0
Isotope cluster parameters: Separation = 1.0 Abundance = 1.0%

Monoisotopic Mass, Odd and Even Electron Ions
6 formula(e) evaluated with 1 results within limits (all results (up to 1000) for each mass)

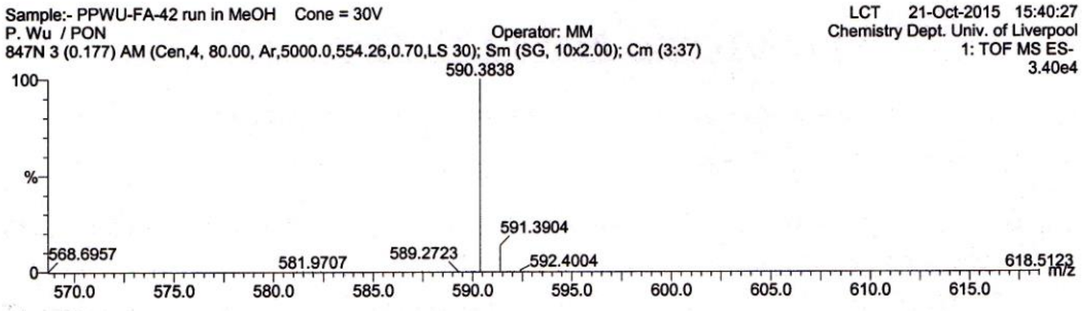

Minimum:	30.00				-30.0		
Maximum:	100.00		4.0	20.0	200.0		
Mass	RA	Calc. Mass	mDa	PPM	DBE	Score	Formula
590.3838	100.00	590.3845	-0.7	-1.3	12.5	1	C37 H52 N O5

HRMS of compound **FA-22**.

Tolerance = 20.0 PPM / DBE: min = -30.0, max = 200.0
Isotope cluster parameters: Separation = 1.0 Abundance = 1.0%

Monoisotopic Mass, Odd and Even Electron Ions
7 formula(e) evaluated with 1 results within limits (all results (up to 1000) for each mass)

Sample:- PPWU-FA-21-1 run in MeOH Cone = 60V LCT 17-Sep-2015 14:02:32
P. Wu / PON Operator: MM Chemistry Dept. Univ. of Liverpool
759N 17 (0.912) AM (Cen,4, 80.00, Ar,5000.0,556.28,0.70,LS 1); Sm (SG, 10x2.00); Cm (5:35) 1: TOF MS ES+
5.31e4

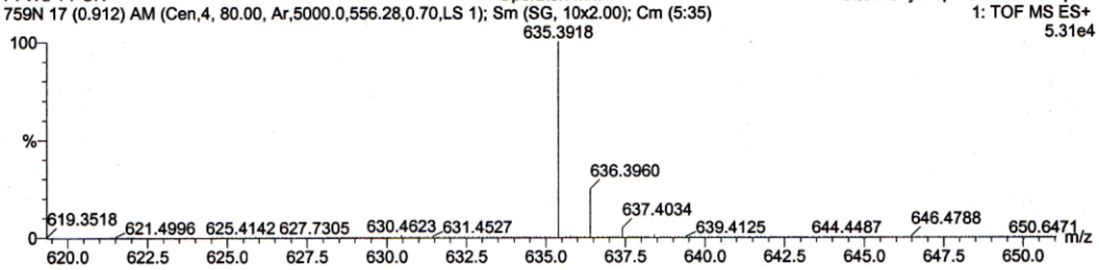

Minimum:	60.00				-30.0		
Maximum:	100.00		4.0	20.0	200.0		
Mass	RA	Calc. Mass	mDa	PPM	DBE	Score	Formula
635.3918	100.00	635.3924	-0.6	-0.9	9.5	1	C37 H56 O7 23Na

HRMS of compound **FA-23**.

Tolerance = 20.0 PPM / DBE: min = -30.0, max = 200.0
Isotope cluster parameters: Separation = 1.0 Abundance = 1.0%

Monoisotopic Mass, Odd and Even Electron Ions
5 formula(e) evaluated with 1 results within limits (all results (up to 1000) for each mass)

Sample:- PPWU-FA-21-2 run in MeOH Cone = 60V LCT 21-Sep-2015 15:47:07
P. Wu / PON Operator: MM Chemistry Dept. Univ. of Liverpool
774N 29 (1.543) AM (Cen,4, 80.00, Ar,5000.0,556.28,0.70,LS 30); Sm (SG, 10x2.00); Cm (5:34) 1: TOF MS ES+
3.65e4

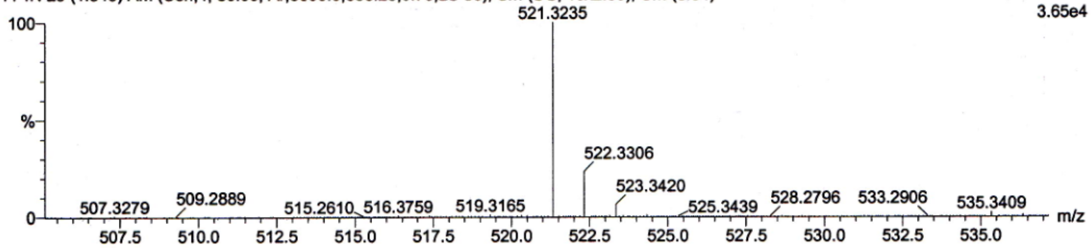

Minimum:	60.00				-30.0		
Maximum:	100.00		4.0	20.0	200.0		
Mass	RA	Calc. Mass	mDa	PPM	DBE	Score	Formula
521.3235	100.00	521.3243	-0.8	-1.5	8.5	1	C31 H46 O5 23Na

HRMS of compound **FA-24**.
